# Exploring the impact of nano-Se and nano-clay feed supplements on interleukin genes, immunity and growth rate in European Sea Bass (*Dicentrarchus labrax*)

**DOI:** 10.1038/s41598-024-53274-y

**Published:** 2024-02-01

**Authors:** Asmaa A. Khaled, Amany M. Shabaan, Saad M. Hammad, Elsayed E. Hafez, Ahmed A. Saleh

**Affiliations:** 1 Animal and Fish Production Department, Faculty of Agriculture (Saba Basha), Alexandria University, Alexandria City, 21531 Egypt; 2 Chemistry Department, Biochemistry Division, Faculty of Science, El-Fayoum University, El-Fayoum, Egypt; 3 Arid Lands Cultivation Research Institute, City of Scientific Research and Technological Applications, New Borg El Arab, Alexandria 21934 Egypt; 4 Present Address: Animal and Fish Production Department, Faculty of Agriculture (Alshatby), Alexandria University, Alexandria City, 11865 Egypt

**Keywords:** Biotechnology, Genetics, Molecular biology, Zoology, Nanoscience and technology

## Abstract

This study aimed to investigate the effects of adding Nano-Selenium (NSe) and Nano-clay (NC) as feed supplements on European Sea Bass (*Dicentrarchus labrax*). Two separate experiments were conducted, one with NC and the other with NSe. Each experiment consisted of four sub-groups with varying concentrations of NC or NSe. The expression levels of five immune-related genes (*TNF-α*, *TNF-β*, *IL-2*, *IL-6* and *IL-12*) were measured using Real-time Quantitative PCR (Rt-PCR) Assay. The results showed an increase in the expression of interleukins (*IL-2*, *IL-6* and *IL-12*) and pro-inflammatory cytokines (*TNF-α* and *TNF-β*) after exposure to NC and NSe**.** *TNF-α* gene expression was significantly higher with both 1 mg and 10 mg concentrations of NC and NSe. *TNF-β* gene expression was highest with the 5 mg concentration of NC. The concentrations of 1 mg and 10 mg for NC, and 1 mg, 5 mg, and 10 mg for NSe, led to the highest (*p* < 0.05) levels of *IL-2* expression compared to the control. Similar trends were observed for *IL-6* and *IL-12* gene expression**.** Understanding the impact of these concentrations on gene expression, growth rate, biochemical indices, and antioxidant status can provide valuable insights into the potential applications of NC and NSe supplements on European Sea Bass.

## Introduction

Fish is considered a vital part of the human diet all over the world because of the high bioavailability of micronutrients^1^. It also represents about 17% of the global population's consumption of animal protein^2^. So, the participation of aquaculture in the overall food supply is huge to solve the universal deficiency problem of white protein. Aquaculture, as the fastest-growing, food sector holds promise for food security and offers new job opportunities^3^. In this regard, feeding aquatic animals a well-balanced diet is essential to felicitous and sustainable aquaculture^4,5^. The aquatic feed should include the optimum requirements from vitamins and minerals alongside carbohydrates, lipids, protein^6^, and antioxidants such as flavonoids and phenolic acids (polyphenols). Where all the above-mentioned additives by specific ratio have shown beneficial effects on fish performance and immunity in aquaculture^7^. Additionally, the presence of trace minerals in aquatic feed improves the physiological and metabolic, characteristics in the organism's body^8^.

On the other side, there are new trends to investigate the potential use of phytoestrogens as a safe alternative to synthetic hormones in aquaculture for stimulating growth and enhancing reproductive features in fish, where phytoestrogens have positive effects on aquatic animal performance, behaviours, and some reproductive features, they should be carefully managed due to the possibility of negative impacts on fish production, reproduction, and behaviours under controlled conditions^9^.

Furthermore, feed supplements are the most familiar administration methods in aquaculture and several supplements are able to boost the immune system of fish and regulate the severity of infections^8^, by affecting several candidate genes, such as; *IL-2*, *IL-6*,* IL-12*, *TNF-α* and *TNF-β*^10–13^.

The European Sea Bass (*Dicentrarchus labrax*) is a commercially valuable fish species known for its economic importance. European Sea Bass is an important cultured fish species in the Mediterranean area with a realizable commercial value^14^. Egypt is one of the leading producers of farmed European Sea Bass, with 24,914 MT in 2018, accounting for 11.5% of the total aquaculture production^15^.

The immune system of fish plays a critical role in combating pathogens and maintaining overall health^16,17^. Interleukins (*ILs*) are key regulators of immune responses, with *IL-2*, *IL-6* and *IL-12* being extensively studied in fish^18–20^.

*IL-2* is a cytokine involved in the proliferation and activation of T lymphocytes^19^. The expression of *IL-2* and *IL-2R* have been observed in various tissues of fish, including the spleen, gut, and gills^19,21^. Studies have suggested that *IL-2* plays a crucial role in the adaptive immune response and fish health^19^. While, *IL-6* is a multifunctional cytokine involved in inflammation, hematopoiesis, and immune regulation^16,22^. *IL-6* and *IL-6R* have been found in fish tissues, including the liver, kidney and gut^23,24^. The *IL-6* pathway is known to modulate innate immune responses in fish, influencing phagocytosis, complement activation and antimicrobial peptide synthesis^20^.

Several studies have investigated the expression and role of *IL-2, IL-2R* and *IL-12* in Gilthead Sea Bream (*Sparus aurata*) and Teleost fish^18–20^. For instance, Secombes et al.^25^ reported upregulated *IL-2* expression upon bacterial challenge in the spleen of Atlantic bluefin tuna (*Thunnus thynnus*), suggesting its involvement in the immune response. Furthermore, Zhao et al.^26^ demonstrated that stimulation of splenic leukocytes with recombinant *IL-2* led to increased lymphocyte proliferation. These findings highlight the importance of *IL-2* in the immune system of fishes. Simona et al.^27^ reported that *IL-6* and *IL-6R* in European Sea Bass has revealed their significant roles in immune regulation. Betül et al.^28^ demonstrated that *IL-6* expression was induced in the liver and kidney of European Sea Bass upon pathogenic challenge. Additionally, activation of the *IL-6* pathway was associated with the upregulation of acute-phase proteins, indicating its involvement in the immune response.

On the other side, the pro-inflammatory cytokines, such as; Tumour-necrosis-factor alpha (*TNF-α*) and *TNF-β*, as well as *ILs* genes are important mediators of inflammatory reactions and orchestrators of the immune system in the vertebrate^29–31^. *TNF-α* is a pleiotropic pro-inflammatory cytokine, that plays a crucial role in regulating the immune response and maintaining immune system balance. Additionally, it is a key player in apoptosis, cell proliferation, differentiation, and sleep regulation^13^. *TNF-α* and *TNF-β* are expressed initially as a membrane-bound peptide and are then enzymatically cleaved by *TNF* convertase (TACE), also known as *ADAM17*^29–31^.

*TNF* gene has been identified and characterized in a variety of invertebrate and vertebrate species; planarians, molluscs, and arthropods^32^. Among fish species, *TNF-α* has been extensively studied in salmonids^33^, carp fish^34^, bluefin tuna^35^ and channel catfish^36^.

Adding Nano-Selenium (NSe) to the diet of European Sea Bass has been suggested as a strategy to enhance the fish's immune response^37,38^. In this aspect, Se supplementation increased the expression of *IL-2* and *IL-6* genes in the gills of European Sea Bass, indicating the potential immunomodulatory effects of NSe. Nano-clay (NC) has also shown promise as a feed supplement for improving immune responses in fish^39,40^. These findings suggest that NC can enhance immune functions, potentially improving disease resistance in fish. Se is a fundamental trace element in fish diets that improves growth performance, immunocompetence, and antioxidative status and helps the body to resist viral infection^41–43^. Se is a component of the enzyme glutathione peroxidase (GPx), which protects the cell membrane from Oxidative damages^8^. Administration of dietary selenium has been reported to enhance growth performance and immunological function in various fish species^44^.

Natural clays are crystalline alumino-silicates characterized by their ability to exchange cations without major changes in structure. They can adsorb toxic by-products of digestion, reducing the accumulation of harmful substances and thereby decreasing the risk of developing diseases. Furthermore, when added to animal diets, they have been observed to increase the profitability of livestock production by enhancing body weight gain^45^. Dietary supplementation with natural clays showed body gain when compared with those fed diet without clay supplementation^46^. Clay is a good choice for making high-performance composite materials because it is cheap and easy to find in nature.

Dietary supplementation of micronutrients to fish in the form of nanoparticles enhances fish productivity and growth performance^47^. Nano-supplementation of micronutrients offers several advantages, such as greater bioavailability, easy absorption, enhanced utilization, and promoting cellular functions. In this aspect, Afifi et al.,^38^ investigated the effect of a nano-coating containing watercress essential oil (EO) and nano essential oil (NEO) on the shelf life of pacific white shrimp stored at refrigerator temperature. The results showed that the nano-coating with NEO reduced oxidative spoilage, lowered microbial load, and increased the shelf life of the shrimp, making it suitable for human consumption.

Recently, Selenium nanoparticle has been utilized due to its high- level bioavailability and low malignancy when fed to fish in adequate quantities^48^. Additionally, NSe improved the growth, feed utilization, and antioxidant defence capacity of numerous cultured fish^49^. At the same time, it was found that dietary supplementation with NC 30 g /kg enhances the growth performance and blood parameters of Nile tilapia fish when compared with a fish-fed diet without any supplementation^50^. Also, Li et al.,^51^ confirmed that dietary NSe supplementation in rainbow-trout (*Oncorhynchus mykiss*) alleviated heat stress-induced intestinal damage by promoting antioxidant enzyme activity, enhancing protein repair, alleviating inflammatory responses, and restoring intestinal microbiota composition. NSe improved intestinal villus height, muscularis thickness, goblet cell number, tight junction protein expression, and reduced the expression of pro-inflammatory cytokines and malondialdehyde content.

Consequently, the present study aimed to investigate the potential of immune-related genes, growth rate, biochemical indices and antioxidant status by adding NSe and NC as Feed Supplements in European Sea Bass. The results of this study could be very useful and applicable to European Sea Bass farmers.

## Results

### Growth performance

The data from Table 1 illustrate the average weight, total and daily gain, and specific growth rate (SGR) of fish in various experimental groups over different time-periods. In the NC experiment, it was observed that fish fed with 5 mg NC/kg diet consistently recorded the highest fish weight, total and daily gain, and SGR values (*p* < 0.05) compared to other groups. Conversely, fish fed with 10 mg NC/kg diet exhibited the lowest weights. Additionally, in the NSe experiment, the fish that were fed with 1 mg NSe/kg diet showed the highest weight, total and daily gain, and SGR values (*p* < 0.05), followed by those fed with 5 mg NSe/kg. The control (NS0) and NS10 groups displayed the lowest weights throughout the experimental period.

**Table 1:** Mean ± Standard Deviation (SD) of fish weights, total gain, daily gain specific growth rate (SGR), and feed conversion ratio (FCR) observed in different experimental groups over the course of the study.

Periods	Groups	Fish weight/days						Total gain (g)	Daily gain (g)	SGR (g)	FCR	Mortality (%)
		Initial weight (g)	The weights during the experiment (g)				Final weight (g)					
		1	15	30	45	60	75					
Acclimatization Period												9.17%
Experiment Period	NC0	12.80 ± 0.64^a^	14.23 ± 0.08^ab^	16.43 ± 0.05^a^	18.40 ± 0.58^a^	21.20 ± 0.08^ab^	24.30 ± 0.83^b^	11.50 ± 0.20^b^	0.153 ± 0.01^b^	0.371 ± 0.01^ab^	2.70^a^	0.00%
	NC1	12.80 ± 0.73^a^	13.90 ± 0.02^ab^	16.03 ± 0.98^ab^	18.03 ± 0.98^a^	20.90 ± 0.74^b^	24.13 ± 0.50^b^	11.33 ± 0.47^b^	0.151 ± 0.01^b^	0.367 ± 0.02^b^	2.54^b^	
	NC5	12.80 ± 0.66^a^	14.33 ± 0.22^a^	16.57 ± 1.09^a^	18.77 ± 1.36^a^	21.83 ± 0.46^a^	25.13 ± 1.45^a^	12.33 ± 0.43^a^	0.164 ± 0.02^a^	0.390 ± 0.03^a^	2.33^c^	
	NC10	12.80 ± 0.62^a^	13.60 ± 0.96^b^	15.63 ± 0.56^b^	17.87 ± 0.87^a^	20.17 ± 0.02^c^	23.30 ± 0.99^c^	10.50 ± 0.32^c^	0.140 ± 0.01^c^	0.346 ± 0.03^c^	2.48^b^	
	NSe0	26.00 ± 0.75^a^	28.67 ± 0.25^a^	32.50 ± 0.72^a^	36.23 ± 0.59^b^	38.80 ± 0.152^c^	42.60 ± 0.99^c^	16.60 ± 0.33^c^	0.221 ± 0.01^c^	0.285 ± 0.09 c	2.81^a^	
	NSe1	26.00 ± 0.53^a^	29.17 ± 0.72^a^	32.67 ± 0.09^a^	36.60 ± 0.48^ab^	39.97 ± 0.92^ab^	45.23 ± 1.54^a^	19.23 ± 0.27^a^	0.256 ± 0.02^a^	0.320 ± 0.01^a^	2.18^c^	
	NSe5	26.00 ± 0.61^a^	29.10 ± 0.80^a^	33.13 ± 1.10^a^	37.13 ± 0.94^a^	40.47 ± 1.69^a^	43.90 ± 0.61^b^	17.90 ± 0.29^b^	0.238 ± 0.01^b^	0.303 ± 0.04^b^	2.33^b^	
	NSe10	26.00 ± 0.69^a^	29.10 ± 0.82^a^	32.60 ± 1.09^a^	36.40 ± 0.34^b^	39.43 ± 2.66^bc^	42.93 ± 0.53^bc^	16.93 ± 0.13^bc^	0.225 ± 0.01^bc^	0.290 ± 0.02^bc^	2.46^b^	

Means ± SD followed by the same letters within a column are not significantly different according to Tukey's Studentized Range (HSD) test at 0.05 level of probability. *NC* Nano-clay, *NSe* Nano-Selenium, *FCR* feed conversion ratio, *SGR* specific growth rate.

### Biochemical indices

In the NC experiment, there was a significant decline in activities of both Alanine Transaminase (ALT) and Aspartate Transaminase (AST) for fish fed on 1 and 5 mg NC/kg diets and a high significant increase in AST for fish fed on 10 mg NC/kg diet compared to control. Also, a significant increase in urea level in fish fed on a 5 mg NC/kg diet compared to control (*p* ≤ 0.05) as presented in Table 2. While there was not any change observed in creatinine concentration within all treated and control groups.

**Table 2:** Biochemical Indices of European seabass fed with test diets for 75 days.

Groups	ALT (U/l)	AST (U/l)	Urea (mg/dl)	Creatinine (mg/dl)
Nano-clay experiment				
NC0 group	12.33 ± 1.52^a^	32.02 ± 3.00^a^	14.02 ± 3.00^a^	0.40 ± 0.16
NC1 group	6.33 ± 1.15^b^	18.70 ± 3.50^b^	17.01 ± 1.00^a^	0.34 ± 0.045
NC5 group	5.17 ± 0.76^b^	10.72 ± 1.51^bc^	19.33 ± 2.5^b^	0.32 ± 0.056
NC10 group	12.70 ± 3.2^acd^	44.70 ± 2.08^bcd^	15.34 ± 1.15^ad^	0.33 ± 0.047
Nano-Se experiment				
NSe0 group	10.67 ± 1.15^a^	19.01 ± 2.00^a^	13.01 ± 1.00^a^	0.27 ± 0.05^a^
NSe1 group	11.67 ± 1.51^a^	19.03 ± 1.00^a^	14.07 ± 1.00^a^	0.27 ± 0.08^a^
NSe5 group	7.67 ± 0.58^ac^	19.30 ± 1.50^a^	14.71 ± 1.30^a^	0.47 ± 0.11^bc^
NSe10 group	33.5 ± 3.20^bcd^	41.7 ± 3.05^bcd^	21.03 ± 2.00^bcd^	0.50 ± 0.11^bcd^

*ALT* Alanine Transaminase, *AST* Aspartate Transaminase, *NC* Nano-clay, *NSe* Nano-Selenium.

NSe treated groups showed highly significant elevation (*p* ≤ 0.01) in urea and creatinine levels in the serum of fish fed on a 10 mg NSe/kg diet. Also, fish fed on 10 mg NSe/kg diets exhibited the highest values of ALT and AST activities as compared to other experimental groups. No significant differences were found in serum AST, ALT and urea in fish fed on 1 and 5 mg NSe compared to control other treatments as presented in Table 2.

### Antioxidative response

Serum Superoxide Dismutase (SOD), Glutathione Peroxidase (GPX) and Catalase (CAT) activities are significantly highest in fish fed on a 5 mg NC/kg diet and fish fed on a 1 mg NSe/kg diet than fish fed on the basal diets (control) as presented in Table 3. While the level of Malondialdehyde (MDA) is significantly lowered (*p* < 0.05) by fish fed on 5 mg NC/ kg and that fed on 1 mg NSe/kg (lowest level) (*p* < 0.05). Further, SOD, GPX and CAT activities had lower values in the group of fish fed on 10 mg NC and 10 mg NSe/kg than fish fed on other treated diets (1 mg or 5 mg/kg), whereas MDA was increased to its highest value in the group of fish fed on 10 mg NSe/kg and 10 mg NC/kg diet (*p* < 0.05) (Table 3).

**Table 3:** Enzymatic antioxidant activities of European seabass fed with test diets for 75 days.

Groups	MDA (IU/I)	SOD (IU/I)	GPX (IU/I)	CAT (IU/I)
Nano-clay experiment				
NC0 group	9.27 ± 0.56^a^	1.41 ± 0.03^a^	3.42 ± 0.32^a^	549 ± 17.00^a^
NC1 group	7.45 ± 0.07^b^	1.59 ± 0.06^a^	3.51 ± 0.28^a^	577.70 ± 18.18^a^
NC5 group	4.90 ± 1.00^bc^	1.81 ± 0.04^b^	4.73 ± 0.19^bc^	587.30 ± 14.04^b^
NC10 group	10.93 ± 0.72^acd^	1.32 ± 0.34^ac^	1.95 ± 1.04^bcd^	431.33 ± 12.01^bcd^
Nano-Se experiment				
NSe0 group	8.91 ± 0.88^a^	1.22 ± 1.00^a^	2.60 ± 0.16^a^	581 ± 5.00^a^
NSe1 group	5.70 ± 0.5^b^	2.25 ± 0.24^b^	3.38 ± 0.045^b^	634 ± 7.41^b^
NSe5 group	6.68 ± 0.45^b^	1.83 ± 0.17^bc^	2.77 ± 0.30^ac^	594 ± 17.51^ac^
NSe10 group	11.72 ± 0.21^bcd^	1.37 ± 0.30^acd^	2.62 ± 0.33^a,d^	551 ± 7.51^bcd^

*MDA* Malondialdehyde, *SOD* Activities of serum superoxide dismutase, *GPX* Glutathione peroxidase, *CAT* Catalase, *NC* Nano-clay, *NSe* Nano-Selenium.

### RAPD and differential display study

Four different interlock primers and RAPD arbitrary primer were used in the DD-PCR reactions to study the presence or absence of genes as a result of different treatments (NC or NSe). RAPD employs arbitrary primers to amplify DNA fragments, generating a range of bands that represent different regions of the genome. The presence or absence of specific bands can indicate the presence or absence of certain genes or genetic variations^52,53^.

**Figure 1:**
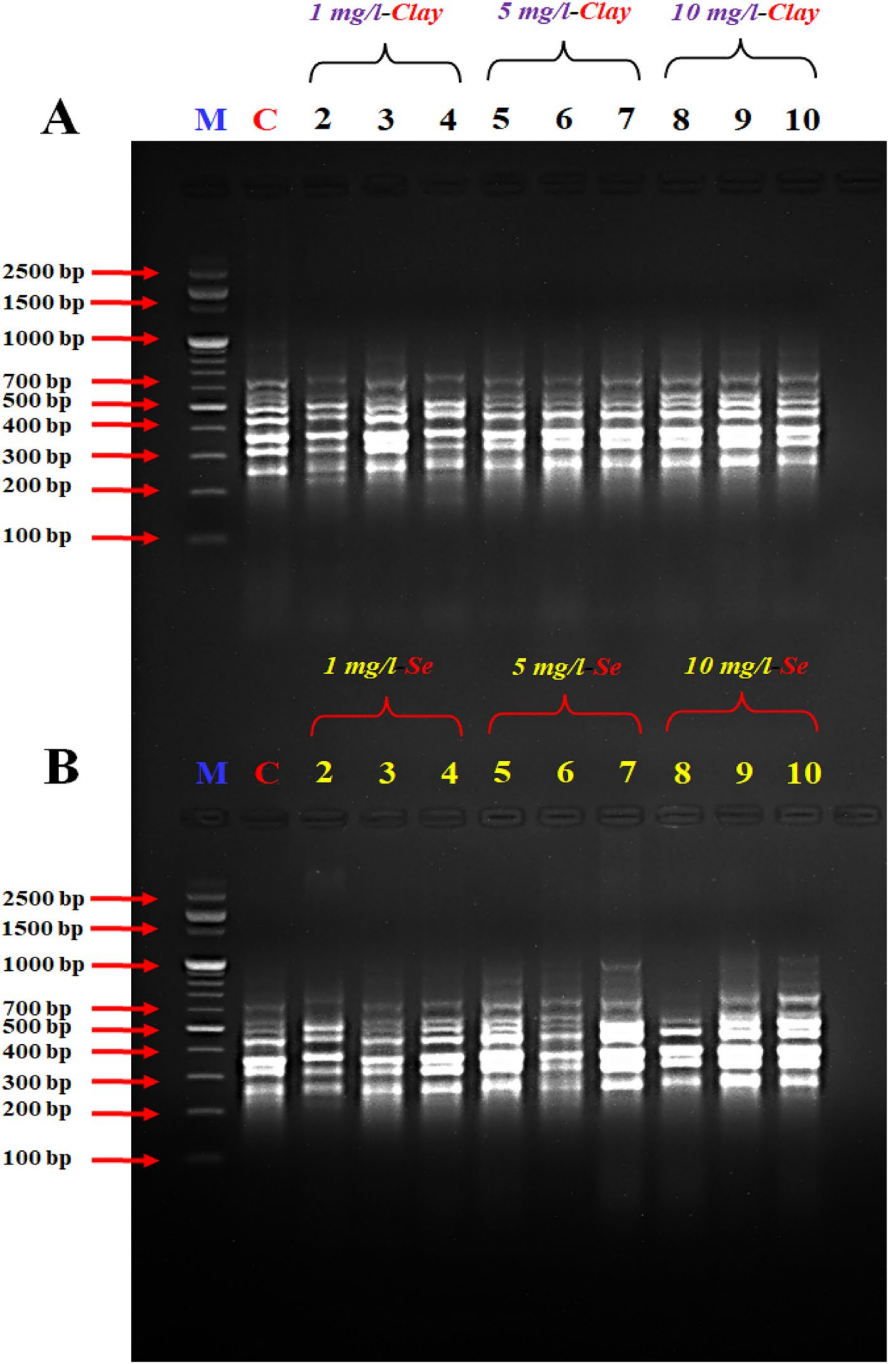
DD- PCR for muscle tissue of fish fed on different concentrations of nano-clay (**A**) and nano-Se (**B**) using RAPD primer *A2*. M: represent DNA marker, Lanes 1: Control treatment (-ve), Lanes 2–4: represents fish supplemented with nano-clay or nano-Se 1 mg/kg diet, Lanes 5–7: represents fish supplemented with nano-clay or nano-Se 5 mg/kg diet, Lanes 8–10: represents fish supplemented with nano-clay or nano-Se 10 mg/kg diet.

**Figure 2:**
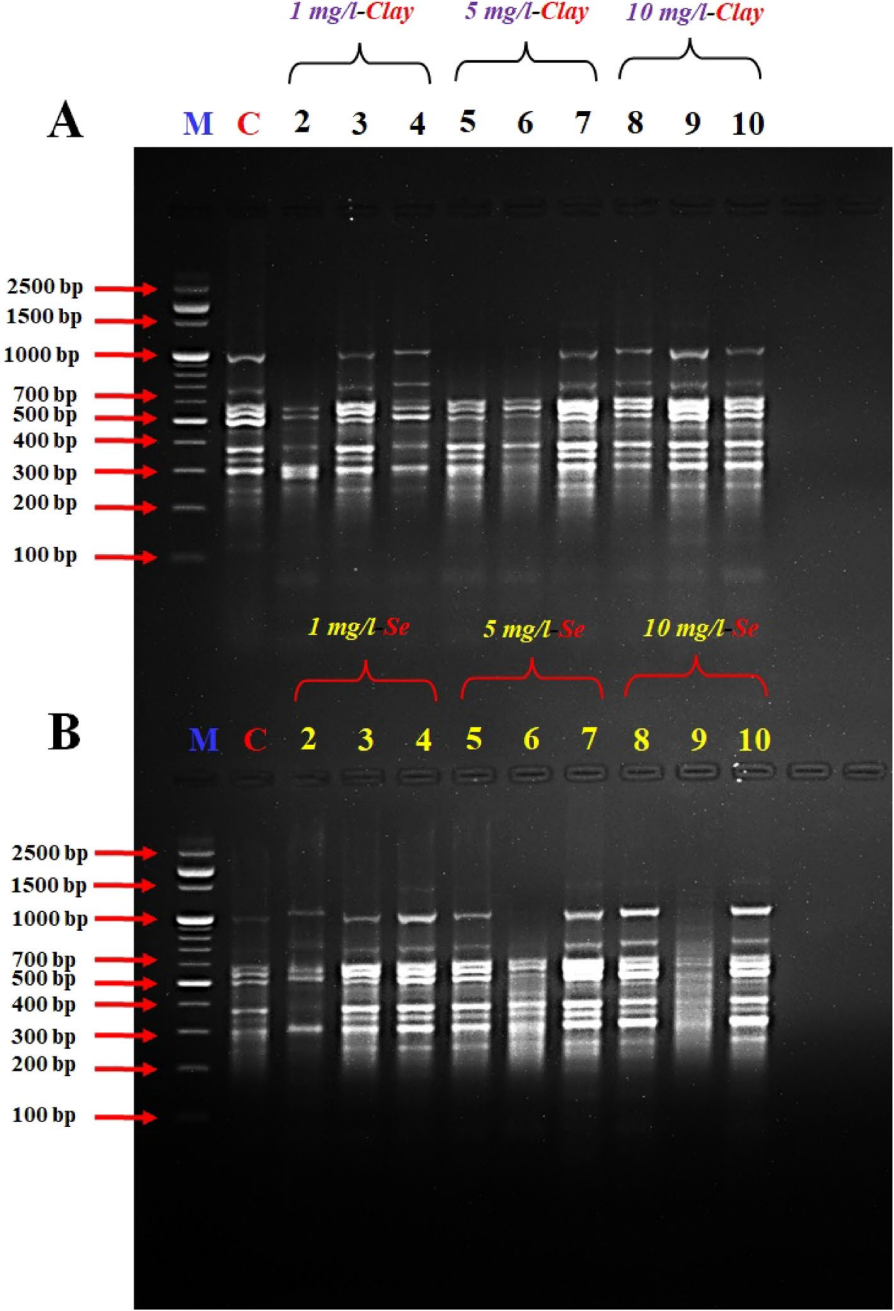
DD-PCR for muscle tissue of fish fed on different concentrations of nano-clay (**A**) and nano-Se (**B**) using primer Interleukin-2 (*IL-2*). M: represent DNA marker, Lane 1: Control treatment (-ve), Lanes 2–4: represent fish supplemented with nano-clay or nano-Se 1 mg/kg diet, Lanes 5–7: represent fish supplemented with nano-clay or nano-Se 5 mg/kg diet, Lanes 8–10: represent fish supplemented with nano-clay or nano-Se 10 mg/kg diet.

**Figure 3:**
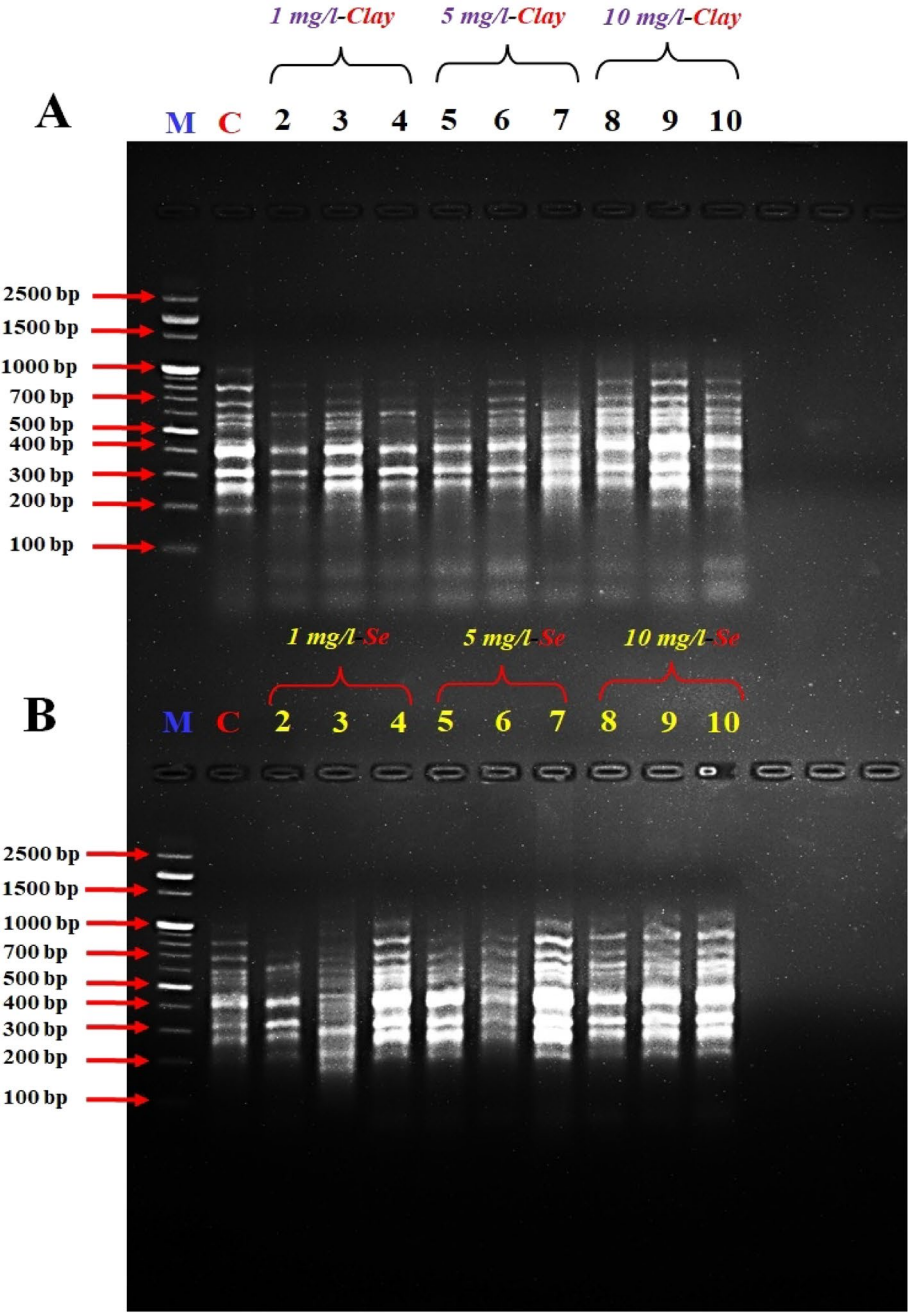
DD- PCR for muscle tissue of fish fed on different concentrations of nano-clay (**A**) and nano-Se (**B**) using primer Interleukin-6F (*IL-6F*). M: represent DNA marker, Lane 1: Control treatment (-ve), Lanes 2–4: represent fish supplemented with nano-clay or nano-Se 1mg/kg diet, Lanes 5–7: represent fish supplemented with nano-clay or nano-Se 5mg/kg diet, Lanes 8–10: represent fish supplemented with nano-clay or nano-Se 10mg/kg diet.

**Figure 4:**
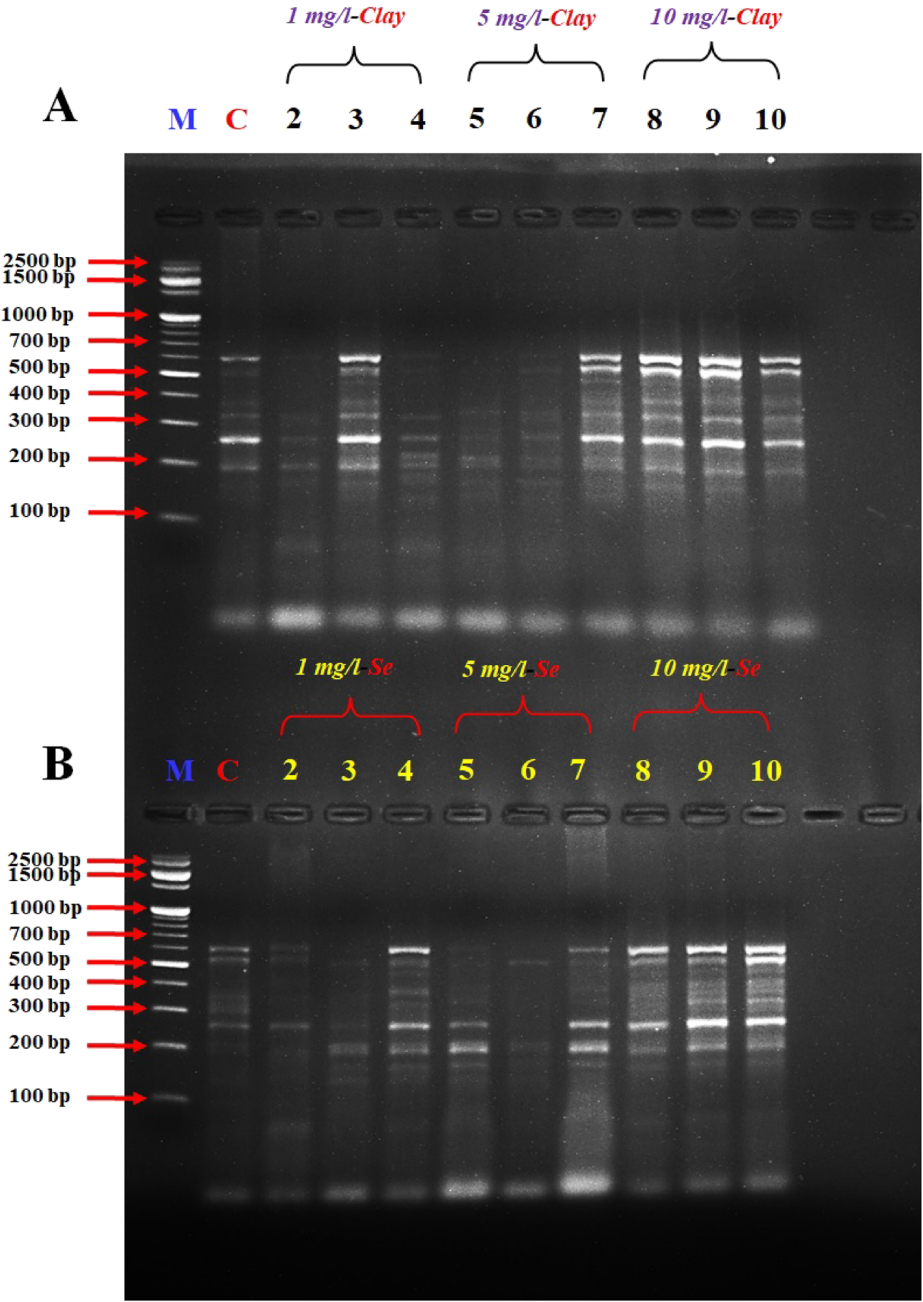
DD-PCR for muscle tissue of fish fed on different concentrations of nano-clay (**A**) and nano-Se (**B**) using primer Interleukin-6R (*IL-6R*). M: represent DNA marker, Lane 1: Control treatment (-ve), Lanes 2–4: represent fish supplemented with nano-clay or nano-Se 1 mg/kg diet, Lanes 5–7: represent fish supplemented with nano-clay or nano-Se 5 mg/kg diet, Lanes 8–10: represent fish supplemented with nano-clay or nano-Se 10 mg/kg diet.

**Figure 5:**
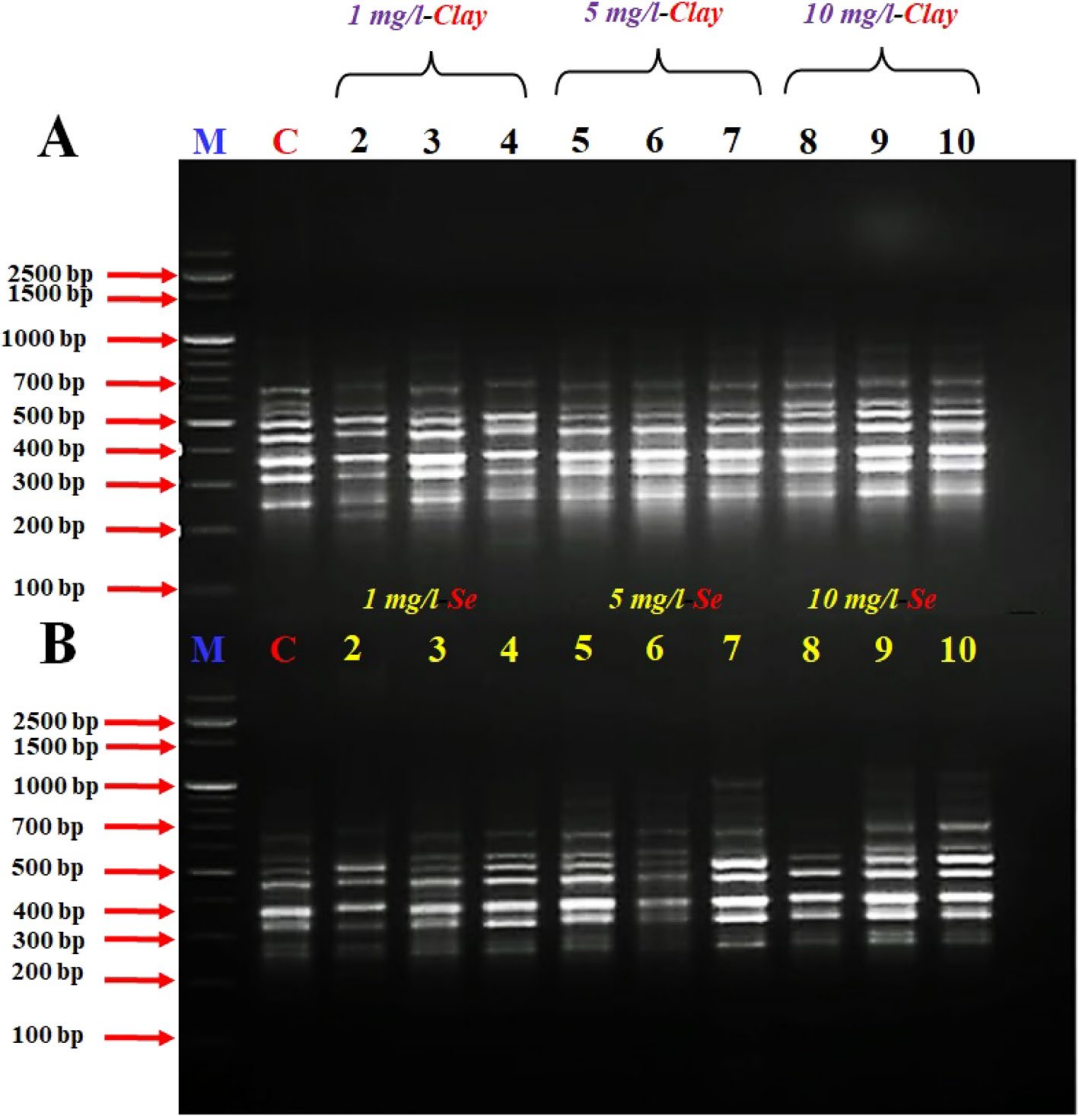
DD- PCR for muscle tissue of fish fed on different concentrations of nano-clay (**A**) and nano-Se (**B**) using primer Interleukin-12R (*IL-12R*). M: represent DNA marker, Lane 1: Control treatment (-ve), Lanes 2–4: represent fish supplemented with nano-clay or nano-Se 1 mg/kg diet, Lanes 5–7: represent fish supplemented with nano-clay or nano-Se 5 mg/kg diet, Lanes 8–10: represent fish supplemented with nano-clay or nano-Se 10 mg/kg diet.

In this study, four different interlock primers and RAPD arbitrary primer were used in DD-PCR reactions to analyse the presence or absence of genes under different treatments (NC or NSe). The observed common bands between control and treated samples in Figs. 1, 2, 3, 4 and 5 suggest that some genetic elements were not affected by the treatments.

The variation in the occurrence and density of genes was examined by analysing the amplified cDNA bands obtained from the examined samples with muscle DNA. Numerous polymorphic (varying among samples) and monomorphic (common in all samples) bands were observed, indicating genetic diversity in the studied groups.

The size range of the obtained DNA bands (between 200 and 1000 bp) suggests the presence of different-sized fragments, potentially indicative of different genes or genetic variations. The presence of these bands in both experiments indicates that there were both up-regulated (turned on) and down-regulated (turned off) genes in the different treatment groups.

Primers *IL-2*, *IL-6F* and *IL-6R* succeeded to differentiate between the examined muscles of NC and NSe treatments, the variety between the examined samples was due to the presence of a high number of induced or suppressed genes when compared to the control (Figs. 2, 3, 4).

The results revealed that the band patterns obtained by these primers differ widely. Consequently, several bands were observed with the primer A2 from 250 to 700 bp for NC experiment, while different bands were observed from 250 to 1000 bp for NSe experiment, in the studied fish which tested by primer *A2* (Fig. 1). Concerning primer *A2*, a specific band/gene (1000 bp) has been observed in the concentrations of 5 and 10 mg NSe compared to the control and other treatments (Fig. 1). DD-PCR obtained from primer *IL-2* showed different band patterns between the NC and NSe treatments especially (5 and 10 mg), and control fish samples. A specific band (800 bp) were observed in the NSe sub-groups but disappeared in the control group (Fig. 2). As for *IL-6F*, other specific bands/genes of 210 and 1000 bp were observed in all NSe sub-group samples but were not presented in the control group (Fig. 3). Regarding *IL-6R*, one specific band/gene of 200 was observed in NSe sub-group samples but was not presented in the control group (Fig. 4). With regard to *IL-12R*, two specific bands (genes) of 800 and 1000 bp were observed in 5 and 10 mg NSe sub-group samples but were not presented in the 1 mg NSe and control groups (Fig. 5).

### Quantitative real-time PCR (QRT-PCR)

**Figure 6:**
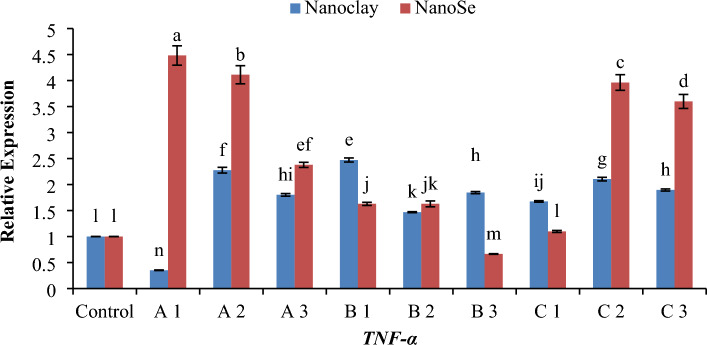
Histogram of the quantitative estimation for Tumour-necrosis-factor alpha (*TNF-α*) gene expressions in fish samples with nano-clay and nano-Se. (**A**) 1 mg/l, (**B**) 5 mg/l and (**C**) 10 mg/l. a–n; letters with a different superscript on the columns are Significant differences (*p* < 0.05).

**Figure 7:**
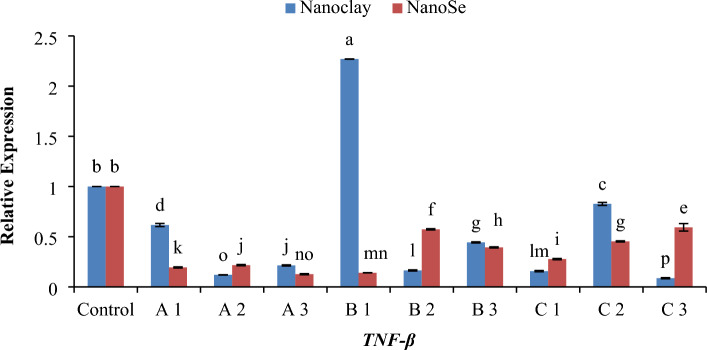
Histogram of the quantitative estimation for Tumour-necrosis-factor Beta (*TNF-β*) gene expressions in fish samples with nano-clay and nano-Se. (**A**) 1 mg/l, (**B**) 5 mg/l and (**C**) 10 mg/l. a–p; letters with a different superscript on the columns are Significant differences (*p* < 0.05).

**Figure 8:**
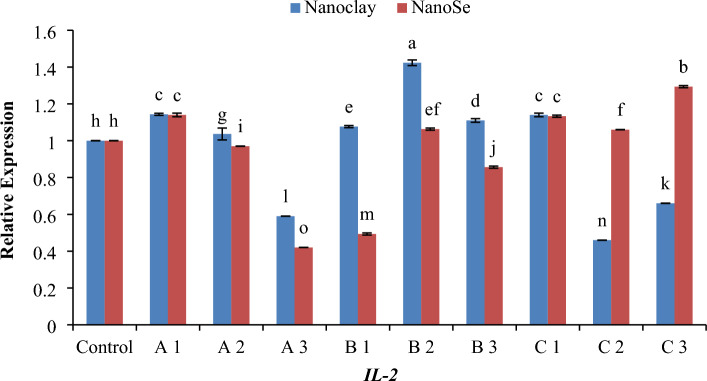
Histogram of the quantitative estimation for Interleukin-2 (*IL-2*) gene expressions in fish samples with nano-clay and nano-Se. (**A**) 1 mg/l, (**B**) 5 mg/l and (**C**) 10 mg/l. a–o; letters with a different superscript on the columns are Significant differences (*p* < 0.05).

**Figure 9:**
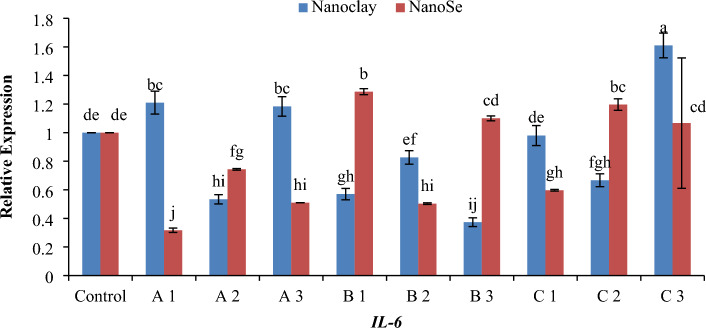
Histogram of the quantitative estimation for Interleukin -6 (*IL-6*) gene expressions in fish samples with nano-clay and nano-Se. (**A**) 1 mg/l, (**B**) 5 mg/l and (**C**) 10 mg/l. a–j; letters with a different superscript on the columns are Significant differences (*p* < 0.05).

**Figure 10:**
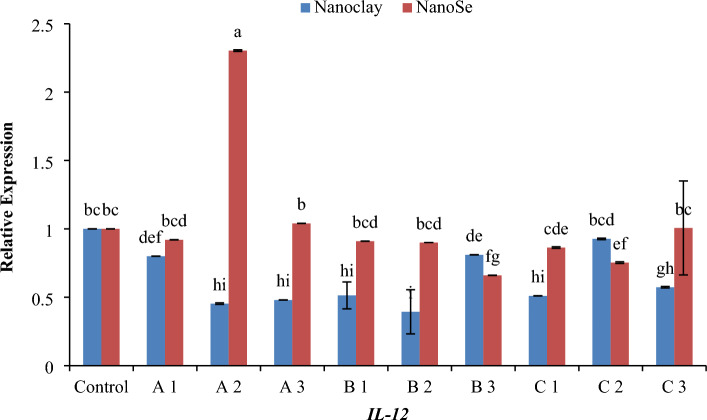
Histogram of the quantitative estimation for Interleukin -12 (*IL-12*) gene expressions in fish samples with nano-clay and nano-Se. (**A**) 1 mg/l, (**B**) 5 mg/l and (**C**) 10 mg/l. a–i; letters with a different superscript on the columns are Significant differences (*p* < 0.05).

Real-time PCR (RT-PCR) was used to detect the relative amounts of mRNA for related protein genes of fish samples. For gene expression quantification, we used the comparative Ct method. First, gene expression levels for each sample were normalized to the expression level of the *β-actin* housekeeping (standard) gene within a given sample (ΔCt); the difference between the treated groups (Fed on NC or NSe with different concentrations) compared to the control was used to determine the ΔΔCT. The log2 ΔΔCT gave the relative fold change in the gene expression of the test versus the control condition. The relative changes in mRNA transcript levels for the examined defence genes *TNF-α*, *TNF-β*, *IL-2*, *IL-6* and *IL-12* were presented in Figs. 6, 7, 8, 9 and 10. Most treated groups revealed better expression of studied genes when compared to the control group. Concerning *TNF-α* gene, the concentration of 1 mg NSe recorded the highest level (*p* < 0.05) of the mRNA transcript followed by 10 mg NSe compared to NC and control (Fig. 6). In relation to *TNF-β* gene, the concentration of 5 mg of NC exhibited the most elevated level (*p* < 0.01) of mRNA transcription when compared to NSe and the control (Fig. 7). Regarding *IL-2* gene, the concentration of 5 mg of NC exhibited the most elevated level (*p* < 0.05) of mRNA transcription followed by 10 mg of NSe when compared to other treatments and the control (Fig. 8). In terms of the *IL-6* gene, the mRNA transcription was observed to be significantly higher (*p* < 0.05) with a concentration of 10 mg of NC followed by 5 mg of NSe and then 1 mg of NC when compared to the other treatments and the control group (Fig. 9). As for *IL-12* gene, a notable increase in mRNA transcription was observed (*p* < 0.01) with a concentration of 1 mg of NSe, surpassing the levels observed with other treatments and the control group (Fig. 10).

**Figure 11:**
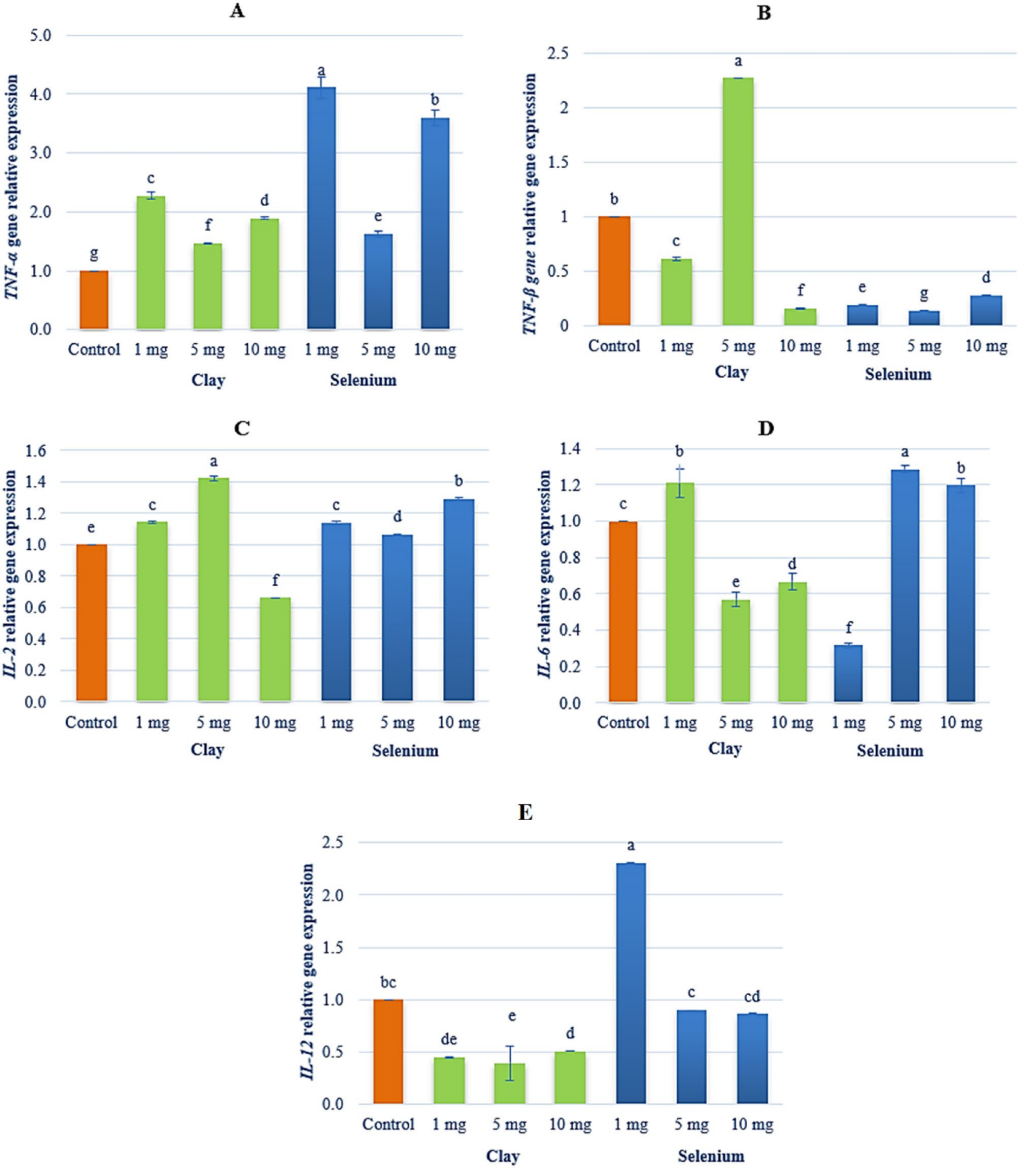
Gene expression of (**A**) Tumour-necrosis-factor Alpha (*TNF-α*), (**B**) Tumour-necrosis-factor Beta (*TNF-β*), (**C**) Interleukin -2 (*IL-2*), (**D**) Interleukin -6 (*IL-6*), (**E**) Interleukin -12 (*IL-12*) in European Sea Bass (*Dicentrarchus labrax*) exposed to Nano-Clay (1, 5 or 10 mg/l; in green colour) and Nano-Se (1, 5 or 10 mg/l; in blue colour) compared to control in orange colour. a–g; letters with a different superscript on the columns are Significant differences (*p* < 0.05).

Figure 11 shows the gene expression of *TNF-α*, *TNF-β*, *IL-2*, *IL-6* and *IL-12* in the European Sea Bass exposed to NC (1, 5 or 10 mg) and NSe (1, 5 or 10 mg). Regarding gene expression, the concentrations of 1 and 10 mg of NC and NSe achieved the highest level of *TNF-α* expression (Fig. 11A). Concerning *TNF-β* gene, the concentration of 5 mg of NC achieved the highest level of gene expression, unlike other concentrations of NC and NSe (Fig. 11B). As for *IL-2* gene, the concentrations of 1 and 10 mg of NC and the concentrations of 1, 5 and 10 mg of NSe recorded the highest level of *IL-2* expression compared to the control (Fig. 11C). With regard to *IL-6* gene, the concentrations of 1 mg of NC and the concentrations of 5 and 10 mg of NSe observed the highest level of *IL-2* expression compared to the control (Fig. 11D). Concerning *IL-12*, the concentration of 1 mg f NSe achieved the highest level of *IL-2* expression compared to the control and other treatments (Fig. 11E).

## Discussion

Recently, dietary supplements with various nano elements become the most important strategy used to achieve optimal production of aquaculture, because the active surface of nano elements facilitates the absorption of the nutrients by the gastrointestinal (GIT) barriers so, improving the growth rate, and inducing the physiological parameters, the immune and antioxidative responses of aquatic animals^37,54^. The present study was performed on European Sea Bass as this species represents one of the most important cultured fish species in Egypt and the Mediterranean region^55^.

The use of NC as fish feed additives recorded a low mortality rate and an improvement in growth rate and biochemical parameters^50^. NSe has high permeability and availability in the fish body so, it enhances the growth rate, biochemical indices and immunity of fish^37^. No studies have been done on the use of NC as a food supplement to enhance the growth rate of European Sea Bass.

The present study showed that the addition of different levels of NC or NSe particles to the diet of European Sea Bass fish for 75 consecutive days may cause improvement in the growth performance of fish according to nano element dose. In the NC experiment, fish fed on a 5 mg NC/kg diet recorded the highest (*p* < 0.05) values of fish weight and growth performance followed by the control group through 15, 30, 45, 60, and 75 days, while fish fed on 10 mg NC/kg diet recorded the lowest weights and growth performance. This result is in agreement with that achieved by Soliman et al*.*^50^ who found that there was a daily weight gain of fish fed with a diet supplemented with NC30 g nano-bentonite/kg diet in rainbow trout (*Oncorhynchus mykiss*). While Ismaila et al*.*^56^ concluded that clay supplemented diet has no adverse effect on the growth rate of fish.

In the NSe experiment the weight and growth performance of fish fed on 1 mg NSe/kg diet recorded the highest value of fish weight followed by that fed on 5 mg NSe/kg. This result is in accordance with many studies that assessed the role of NSe in improving the growth performance of common carp (*Cyprinus carpio*)^57^, European Sea Bass^37^ and Nile tilapia (*Oreochromis niloticus*)^58^. In the present work, the lowest weights were from the control (NS0) and NS10 groups through the experimental period. This can be explained by Lee et al.^59^ who revealed that the level of selenium needed to achieve optimum growth efficiency may vary based on the type of selenium, the time of administration and the experimental technique, as well as fish species. In the present study, the improvement of body weight when fish fed with nano element supplemented diets (NC or NSe) may be due to the valuable effects of these antioxidants on the growth hormone hence the growth performance of fish^60^. NSe also enhances protein digestibility and promote protein synthesis in the intestinal epithelial cells hence improve the growth of fish^61^.

On the other hand, serum biochemical indices provide beneficial diagnostics to monitor the well-being status of cultured fish^62^. The liver is the organ that is responsible for detoxification and metabolism. Plasma transaminase enzymes (AST and ALT) are used to assess liver function as they participate in nitrogen metabolism in cellular tissues, so their high level in plasma may indicate hepatocyte damage due to toxicity^63^.

In the current study, there was a significant decline in the activities of both ALT and AST in fish fed on 1 and 5 mg NC/kg diets. The present findings agree with Soliman et al*.*^50^ who stated that transaminase enzymes were significantly (*p* < 0.05) decreased as affected by NC dietary feed additives of fish. The current results also revealed a highly significant increase in AST in fish fed on a 10 mg NC/kg diet compared to the control. But at all the higher levels of liver enzymes are not always associated with liver injury. In the present results also, fish fed on 10 mg NSe/kg diets exhibited the highest values of ALT and AST activities as compared to other experimental groups (Table 2). No significant differences were found in serum AST and ALT in fish fed on 1 and 5 mg NSe compared to each other or control. These results agree with Ibrahim et al.^64^ who found the lowest activities of plasma ALT and AST in the fish-fed diet containing 0.8 mg NSe/ kg diet as compared with other treatment diets. Also, Abd El-Kader et al.^65^ observed no significant alterations in ALT and AST variables in fish fed with varying levels of Se nanoparticles. According to these results, we conclude that NSe with the level of 1 or 5 mg/kg has a protective effect on liver cells, can scavenge free radicals and may also promote cytokine production.

Concerning the products of nitrogen metabolism blood urea nitrogen (BUN) and creatinine are mainly assessed in the blood of organisms, particularly fish. The level of BUN is similar in various freshwater fish^65^. Creatinine is the end product of creatine metabolism in muscle and is excreted through the kidneys^66^. Consequently, high levels of creatinine in fish blood might be due to muscle injury or kidney disorder that inhibits its excretion.

In the current study, a significant increase in urea level in fish fed on a 5 mg NC/kg diet was observed compared to the control (Table 2). This increase in urea level may be due to the effect of diet, rather than pathological. While there were no changes observed in creatinine concentration within all treated and control groups. The present result is in the same line with that obtained by Soliman et al*.*^50^ who observed a significant decrease in creatinine levels in the NC group as compared to the control. On the other hand, Mascolo et al.^67^ detected an increase in kidney function and accumulation of NC elements in rats, urine and tissues. The current results also showed highly significant elevation (*p* ≤ 0.01) in urea and creatinine levels in the serum of fish fed on a 10 mg NSe/kg diet. These results showed that the aquaculture diet containing high concentrations of NSe may become toxic to aquatic life. A non-significant difference was observed in urea and creatinine levels in fish fed with NSe at 1 or 5 mg/kg compared to the control. This study was in agreement with Ziaei-nejad et al.^68^ who detected non-significant change in creatinine levels in fish fed with different NSe concentrations. Also, Ahmed et al.^69^ concluded that there was an improvement in urea and creatinine levels in fish groups fed on NSe. The current study revealed that dietary NSe supplementation of concentration 1 mg/kg improves liver and kidney function. This improvement might be due to its role in breaking the free radical reactions chain by allowing free radicals to eliminate the hydrogen atom from the antioxidant molecule rather than polyunsaturated fatty acids, resulting in the formation of relatively unreactive radical species that protect kidney tissue from peroxidative damage^69^.

The production of reactive oxygen species (ROS) and release of free radicals is known as oxidative stress, fish have antioxidant enzymes that act as a defence against these ROS^70^, GPX, SOD and CAT are antioxidant enzymes used to inhibit ROS production and protect the body from damage such as damage of the cell metabolism function and structure^71^. SOD and CAT can degenerate ROS and reduce lipid peroxidation injury, also it is essential to estimate the lipid peroxidation level by measuring the malondialdehyde (MDA) level^72^. The failure to degenerate the free radicals results in the impairment of the cell DNA and, thereby, the tissue’s death^73^.

In the present work, SOD, GPX and CAT activities are significantly highest in fish fed on a 5 mg NC/kg diet and fish fed on a 1 mg NSe/kg diet than in fish fed on the basal diets/control (Table 3). This result is in agreement with that obtained by Smirnova et al.^74^ who experimented on male rats that were exposed to NC orally in a dose equal to 1 or 100 mg/kg of body weight, and found that an antioxidant protection system became more active (diene conjugates contents in plasma went down and glutathione peroxidase became more active). At the same time, the antioxidant ability of NSe is related to its role in the synthesis of proteins required for GPX activation. The current study also agrees with the study of Abd El-Kader et al.^64^ who found that SOD, CAT and GPX activities were significantly higher in fish fed varying levels of NSe than fish fed the basal diet and the level of MDA is significantly lowered by dietary NSe supplemented in the diet of European Sea Bass^64^, Nile Tilapia^58^ and Striped catfish^75^.

In the present study, the level of MDA is significantly lowered by fish fed on 5 mg NC/kg and that fed on 1 mg NSe/kg (lowest level) (*p* < 0.05), this result is in agreement with several studies^72,73^ and confirms the effective role of both NC and NSe at certain doses in protecting European Sea Bass against lipid peroxidation. Furthermore, in the present study, SOD, GPX and CAT activities had lower values in the group of fish fed on 10 mg NC and 10 mg NSe/kg than fish fed on other treated diets (1 mg or 5 mg/kg), whereas MDA was increased to its highest value in the group of fish fed on 10 mg NSe/kg and 10 mg NC/kg diet. These results confirm that the increase in the concentration of nano element used as feed additives may give adverse effects, cause oxidative stress and increase lipid peroxidation of fish.

In the molecular part of the current investigation RAPD technique provided insights into the presence or absence of certain genetic elements, it does not directly quantify gene expression levels. However, by comparing the banding patterns between different samples or treatment groups, it is possible to infer potential up-regulation (increased expression) or down-regulation (decreased expression) of genes.

In RAPD, the presence or absence of bands represents the presence or absence of specific DNA fragments or regions in the samples being analysed^52^. If a band is consistently present in treated samples but absent in control samples, this suggests that the corresponding genetic element may be up-regulated in response to the treatment. Conversely, if a band is absent in treated samples but present in control samples, it suggests down-regulation of that genetic element due to the treatment^53^.

However, it is important to note that RAPD does not provide quantitative data on gene expression levels. To confirm and further investigate the up-regulation or down-regulation, additional techniques such as real-time PCR or RNA-sequencing would be necessary. These methods can provide more precise measurements of gene expression levels. When specifying up-regulation or down-regulation, it is important to utilize additional complementary techniques and consider the context of the study. Comparative analysis and functional annotation of the identified bands can help determine the potential roles or functions of the differentially expressed genes. Additionally, referencing relevant studies or databases that provide insights into gene expression patterns in similar systems or organisms can strengthen the interpretation of the observed regulation patterns^76,77^. Therefore, in the current study, to build a complete visualization, reliable results and insight, we used RT-PCR and RT-PCR techniques.

Both DD-PCR and RT-PCR are powerful tools used for investigating gene expression patterns, as well as identifying differentially expressed genes in various organisms^76,77^. Both techniques were employed in the current study.

In the context of scanning interleukin genes *IL-2R*, *IL-6F*, *IL-6R* and *IL-12R* in the European Sea Bass genome, DD-PCR can provide valuable insights into the immune response and overall health of this economically important fish species. The present genes are crucial interleukins involved in the regulation and coordination of immune responses. By using DD-PCR, we have examined the expression profiles of these genes in the tissues. One major advantage of DD-PCR is its ability to identify gene expression changes without prior knowledge of the target sequences. By selectively amplifying differentially expressed genes, DD-PCR allows for the discovery of novel genes or isoforms that may play important roles in immune function and disease resistance^76–78^ in European Sea Bass. Furthermore, DD-PCR can help elucidate the regulatory mechanisms underlying interleukin gene expression^77^. By comparing samples with different gene expression patterns, we identified the potential regulatory elements that control the expression of *IL-2R*, *IL-6F*, *IL-6R* and *IL-12R* genes (Figs. 1, 2, 3, 4, 5) and the full length of these figures are shown in the supplementary file (1): Figs. S1-S5. These genes were successful in differentiating between the muscles of NC and NSe treatments. The variation observed in the examined samples was attributed to the presence of a high number of induced or suppressed genes compared to the control. Each primer showed different band patterns, indicating unique gene expression profiles. For example, with *A2* primer, different bands were observed in the NC and NSe experiments, ranging from 250 to 700 bp and 250 to 1000 bp, respectively. A specific band of 1000 bp was observed in the concentrations of 5 and 10 mg NSe compared to the control and other treatments. Similarly, *IL-2* showed different band patterns between the NC and NSe treatments, with a specific band of 800 bp observed in the NSe sub-groups but not in the control group. *IL-6F* revealed other specific bands of 210 and 1000 bp in all NSe sub-group samples but not in the control group. Additionally, *IL-6R* showed a specific band of 200 bp in NSe sub-group samples but not in the control group. Lastly, with *IL-12R*, two specific bands of 800 and 1000 bp were observed in the 5 and 10 mg NSe sub-group samples but not in the 1 mg NSe and control groups. Understanding the expression patterns and regulatory mechanisms of these interleukin genes is crucial for improving the management and health of European Sea Bass in aquaculture settings^18–20^. Overall, the results demonstrate different gene expression patterns between the NC and NSe treatments, and specific primers were able to identify distinct gene bands associated with these treatments.

Regarding gene expression, the effects of different concentrations of NC and NSe on the mRNA transcript levels of *TNF-α*, *TNF-β*, *IL-2*, *IL-6* and *IL-12* genes have been investigated. The results showed that when it came to the expression of the *TNF-α* gene, a concentration of 1 mg NSe recorded the highest level of mRNA transcription, followed by 10 mg NSe (Fig. 6). These concentrations exhibited significantly higher gene expression compared to NC and the control group. Moving on to the *TNF-β* gene, the concentration of 5 mg NC demonstrated the most elevated level of mRNA transcription, surpassing the levels observed with NSe and the control group (Fig. 7). This elevation in mRNA transcription was found to be statistically significant when compared to the other treatments. In terms of *IL-2* gene, a concentration of 5 mg NC exhibited the highest level of mRNA transcription, followed by 10 mg NSe (Fig. 8). Both concentrations showed significantly elevated gene expression compared to the other treatments and the control group. With regard to *IL-6* gene, the mRNA transcription was observed to be significantly higher with a concentration of 10 mg NC, followed by 5 mg NSe and then 1 mg NC (Fig. 9). These concentrations exhibited significantly higher mRNA transcription compared to the other treatments and the control group. Finally, the findings showed that the mRNA transcript of *IL-12* gene increased notably with a concentration of 1 mg NSe, surpassing the levels observed with other treatments and the control group (Fig. 10). This increase in mRNA transcription was found to be statistically significant. Overall, the findings suggest that different concentrations of NC and NSe have varying effects on the mRNA transcript levels of *TNF-α*, *TNF-β*, *IL-2, IL-6* and *IL-12* genes. The results highlight the potential of NSe, particularly at a concentration of 1 mg, for enhancing the mRNA transcription of certain genes, while NC, particularly at a concentration of 5 mg, exhibited superiority in enhancing the mRNA transcription of specific genes. Further research is warranted to explore the underlying mechanisms and determine the optimal concentrations for maximizing mRNA transcription.

Regarding RT-PCR findings, the data presented in Fig. 11A demonstrates that the concentrations of both 1 mg and 10 mg of NC and NSe exhibit the highest level of *TNF-α* expression. This finding suggests that these particular concentrations have a significant impact on *TNF-α* gene expression. In contrast, when examining the *TNF-β* gene expression depicted in Fig. 11B, it becomes apparent that only the concentration of 5 mg of NC leads to the highest level of gene expression. Interestingly, the other concentrations of NC and NSe do not elicit the same effect. Moving on to *IL-2* gene, Fig. 11C illustrates that the concentrations of 1 mg and 10 mg of NC, along with the concentrations of 1 mg, 5 mg, and 10 mg of NSe, result in the highest level of *IL-2* expression when compared to the control. This indicates that these specific concentrations have a greater impact on *IL-2* gene expression. Similarly, Fig. 11D demonstrates that the concentrations of 1 mg of NC and 5 mg, and 10 mg of NSe lead to the highest level of *IL-6* expression compared to the control. This suggests that these concentrations have a pronounced influence on *IL-6* gene expression. Lastly, the concentration of 1 mg NSe stands out as achieving the highest level of *IL-12* expression when compared to both the control group and other treatments (Fig. 11E). This specifies that this particular concentration plays a critical role in enhancing *IL-12* gene expression. Overall, these findings indicate that different concentrations of NC and NSe have varying effects on the expression levels of *TNF-α*, *TNF-β*, *IL-2*, *IL-6* and *IL-12* genes.

The concept of investigating both mRNA transcription and gene expression in the present study due to the key difference between mRNA transcription and gene expression lies in their scope. mRNA transcription specifically refers to the synthesis of mRNA molecules from a DNA template^79^. It is a localized process that occurs in the nucleus, involving the initiation, elongation, and termination of transcription. Gene expression, however, is a broader term that encompasses all the events involved in the synthesis of a functional protein. It includes not only mRNA transcription but also various additional processes that occur in different cellular compartments. These processes collectively determine the abundance and activity of the final protein product^79,80^.

By understanding the impact of the above concentrations of NC and NSe on gene expression, further research can be conducted to explore their potential applications within medical and biotechnological fields.

## Conclusions

In conclusion, the use of dietary supplements with nano elements, such as NC and NSe, has shown promising results in improving the growth rate and physiological parameters of aquatic animals, specifically European Sea Bass. The addition of NC to the fish diet led to a low mortality rate and improved growth rate and biochemical parameters. On the other hand, NSe enhanced the growth rate, biochemical indices, and immunity of fish. The addition of NC or NSe to the diet of European Sea Bass fish improved their growth performance, with optimal results seen at specific dosage levels. Furthermore, these nano elements had a protective effect on liver cells and were able to scavenge free radicals, thereby promoting cytokine production. In terms of nitrogen metabolism, NC and NSe had varied effects on urea and creatinine levels, with certain concentrations leading to significant changes in these parameters. Additionally, both NC and NSe exhibited antioxidant properties, as evidenced by the increased activities of antioxidant enzymes and decreased levels of lipid peroxidation. Lastly, the examination of gene expression patterns using DD-PCR and reverse transcription PCR revealed that different concentrations of NC and NSe had varying effects on the mRNA transcription. Also, the gene expression of immune-related genes, including *TNF-α*, *TNF-β*, *IL-2*, *IL-6* and *IL-12*. Further research is needed to explore the underlying mechanisms and determine the optimal concentrations for maximizing the beneficial effects of NC and NSe in aquaculture settings.

## Material and methods

### The ethical approval

All fish and sampling procedures in this experiment were supervised and approved by the Institutional Animal Care and Use Committee of the Faculty of Agriculture, Alexandria University, Egypt (No.: AU0821045175). All procedures and experimental protocols were under the Guide for the Care and Use of Agricultural Animals in Research and Teaching, Federation of Animal Science Societies (FASS, 2010) https://www.aaalac.org/about/Ag_Guide_3rd_ed.pdf. Also, the study was carried out in compliance with the ARRIVE guidelines (https://arriveguidelines.org).

### Nano-clay (NC) and Nano-selenium (NSe) preparation

NC used in the present work was prepared and characterized according to Aseel et al.^81^. Egyptian alluvial soil samples were randomly collected from the top 15 cm of the depth of the sugar beet field (*Kafer-Elshekh* governorate, Egypt; GPS: 31.5802538596, 31.1312295632) air-dried, grinded and sieved at < 2 mm. The soil sample was pre-treated with distilled water, sodium acetate (0.5 M) and 30% hydrogen peroxide to remove salts, carbonates and organic debris. Then the obtained NC was characterized using Scanning Electron Microscope, FTIR and X-ray diffraction.

NSe used in this study was prepared biologically according to Zhang et al.^82^ and Chen et al.^83^ with some modification, 1 ml of hydrazine (N_2_H_4_, 50%, v/v) and 0.2 g of selenious acid were put into a Teflon-lined autoclave filed cyanobacteria water extract up to 80% of the total volume, sealed and maintained at 110–180 ◦C for 14 h, and then cooled to room temperature. The black product was filtered off and washed with distilled water and absolute ethanol, and finally dried in air.

### Diet preparation

The basic diet (45% crude protein) was purchased from Aller Aqua for Fish Feed Manufacture, 6 October City-Giza, Egypt (https://www.aller-aqua.com). Formulation of the diet and the approximate composition of the basic diet was conducted according to AOAC^84^, and were shown in Table 4. In the present work, eight experimental diets were prepared to contain NC at rates of (0, 1 mg, 5 mg and 10 mg NC/kg diet) and NSe at rates of (0.1 mg, 5 mg and 10 mg NSe/kg diet). The experimental diets containing either NC or NSe particles were quite mixed separately with the basal diet in the presence of fish oil and water. The prepared diets were pressed using a manufacturing machine (pellets size 1 mm), dried by air at room temperature for 36 h (h) and then stored at 4 °C until used. The diet pellets were prepared according to Royes and Chapman^85^, Gonzalez et al.^86^ and Bermejo-Nogales et al.^87^.

**Table 4:** Formulation and proximate composition of the basal diet.

Ingredient		Chemical composition	
Low-fat fish meal (%)	35	Crude protein (%)	45
Soybean meal (%)	20	Crude fibres (%)	2.6
Corn gluten meal (%)	10	Crude fat (%)	15
Wheat flour (%)	10	Gross energy (kcal/ kg)	4837
Fish oil (%)	5		
Soybean oil (%)	2		
Mono-sodium phosphate (%)	0.5		
Vitamins and minerals mixture (%)	5		
Dl-Methionine/Choline Chloride (%)	2		

### Fish and experimental design

European Sea Bass fish (n = 600) were obtained from a commercial fish farm (GPS; 31.042120, 30.853177). Before the experiment, the fish were left for fifteen days in rounded fibreglass tanks (10 m^3^) for acclimatization, supplied with dechlorinated tap water and aerators, and were fed with basic diet (45% crude protein) as shown in Table 4. After the acclimatization period, the number of fish decreased to 545 fish (mortality = 9.17%). 480 fish have been used for the next step of the experiment. The fish (n = 480) were distributed randomly into eight groups: Four groups of the NC experiment and another four groups for the NSe experiment. Each experimental diet was assayed in triplicate (4 experimental diets × 3 fibreglass tanks) at a stocking density of 20 fish per enclosure (hapa) for the NC experiment and for the NSe experiment. All tanks were supplied with an air compressor.

#### Groups of Nano-Clay (NC) experiment

The initial body weight means of these groups were 12.8 g/ fish. They were classified as follows: (1) NC0 group: was considered as a negative control (NC0) and fed with basal diet during the experimental period (75 days). (2) NC1 group: was fed daily with a diet containing NC (1 mg/kg of basal diet) for 75 days. (3) NC5 group: was fed daily with a diet containing NC (5 mg/kg of basal diet) for 75 days. (4) NC10 group: was fed daily with a diet containing NC (10 mg/kg of basal diet) for 75 days.

#### Groups of Nano-Se (NSe) experiment

The initial body weight means of these groups were 26 g/ fish. They were classified as follows: (1) NSe0 group: was considered as the negative control group and fed with basal diet during the experimental period (75 days). (2) NSe1 group: was fed daily with a diet containing NSe (1 mg/kg of basal diet) for 75 days. (3) NSe5 group: was fed daily with a diet containing NSe (5 mg/kg of basal diet) for 75 days. (4) NSe10 group: was fed daily with a diet containing NSe (10 mg/kg of basal diet) for 75 days.

Fish were fed three times daily at 9:00, 15:00 and 21:00 with each meal over the experimental period. Every day, each glass tank of fish was cleaned and the water was somewhat altered (about 25%). Fish were weighed on the first day, after 15-, 30-, 45- and 75-days during the experiment to calculate growth and health states. The water quality parameters were adjusted at the optimum range for European Sea Bass as follows: Temperature (18.0 ± 0.5 °C), dissolved oxygen (5.1 ± 0.3 mg/L), pH (7.3 ± 0.2) and ammonia (0.03 ± 0.004 mg/L) with a controlled photoperiod (12h lightness:12h darkness). The feed conversion ratio (FCR) was calculated according to Yu et al.^88^. While, total gain, daily gain and specific growth rate (SGR) were calculated according to Peterson et al.,^89^, Islam et al.,^90^ and Alvarado et al.,^91^, using the following formulas;

1 $${\text{Total}}\,{\text{gain}} = {\text{final weight}} - {\text{initial weight}}$$

2 $${\text{Daily gain}} = {\text{total gain}}/{\text{number of days}}$$

3 $${\text{SGR}} = [{\text{log}}\,\left( {{\text{final weight}}} \right) - \log \left( {{\text{initial weight}}} \right)/{\text{number of days}}]*100$$

4 $${\text{FCR}} = {\text{feed consumed}}/{\text{weight gain}}$$

### Blood and tissue sampling

After 75 days (at the end of the Nano-experiments), all fish were anaesthetized using 25 mg/L MS-222 (Syncaine®, USA). Blood samples were withdrawn according to Zang et al.^92^ from the caudal vasculature of three randomly selected fish from each tank using a syringe without anticoagulant, and serum was separated by centrifuging the clotted blood at 3000 rpm/15 min at 4 °C. Isolated sera were stored at -80 °C until further analysis and blood enzyme determination. After blood sampling, portions of muscle tissues were dissected immediately from the same fish and kept in RNA later (Bioshop, Germany) at -80 °C for RNA extraction, genetic analysis and gene expression.

### Biochemical parameters and oxidation assays

Serum enzymatic activity of AST and ALT were assayed using a kit from BIOLABO Co., according to the method of Henry et al.^93^. Renal function biomarkers; Serum Creatinine and Urea were evaluated using a kit from BIOLABO Co., according to assays of Fabiny and Ertingshausen^94^ and Tiffany et al.^95^ respectively.

Activities of SOD, GPX, CAT and MDA concentration were assessed using diagnostic reagent kits following the procedure for the manufacturer (Cusabio Biotech Co., Ltd).

### Gene expression

#### Total RNA Extraction and cDNA Synthesis

Total RNA was extracted from fish muscle samples (control, and three replicas of each treated group) using Total RNA extraction methods according to Chomczynski et al.^96^. The RNAs concentration and purity were measured using Nano-drop (NanoDrop 2000c spectrophotometer, Thermo, USA). RNA was stored at -80 °C in a horizontal deep-freezer (Revco, USA) until use it. Complementary DNA (cDNA) was synthesized by reverse transcribing total RNA in a 20 μL reaction containing the following; 3 μL of total RNA, 5 μL of oligo (-dT) primer (10 pmol/µL), 2.5 μL of dNTP (10 mL), 2.5 μL of buffer (10*x*), 0.3 μL of Reverse Transcriptase (M-MULV Reverse Transcriptase 200 unit/µL, Biolabs, England), and 6.7 μL sterile dH_2_O to final volume of 20 μL. The reaction was mixed gently by shaking and placed in a thermo-cycler (MJ Research, Inc., PTC-100™ Programmable thermal controller, USA). The amplification was programmed as follows: 37 °C for 1.30 h, followed by inactivation at 80 °C for 10 min, and cooling at 4 °C. The newly generated cDNA-RNA hybrid was then stored at -20 °C.

#### Differential display PCR reaction

Four different interleukin primers (*IL*) and RAPD arbitrary primer (*A2*); *A2*^97^, *IL-2R*^98^, *IL-6F*, *IL-6R*^99^ and *IL-12R*^100^ were used to scan the mRNA genes in fish samples as shown in Table 5. The amplified cDNA was used as a template for the differential display reaction. The PCR reaction component was performed as10μL master mix, 1 μL cDNA (50 ng), 5 μL from primer (10 pmol ⁄μL) and sterile dH_2_O up to volume 20 μL. The PCR program was applied as follow; initial denaturation at 95 °C for 3 min; 40 cycles of 94 °C for 30 s; annealing at 51 °C for 30 s; extension at 72 °C for 1min. Final extension step at 72 °C for 5 min. A 5 μL of PCR products were separated on 2% (w/v) agarose gel electrophoresis in 0.5 × TBE buffer. The molecular length of the PCR product was estimated using DNA molecular length marker. Finally, the gel was photographed by Gel Documentation System (Chemi.Doc™ XRS + with Image Lab™ Software, BIO-RAD, USA).

**Table 5:** The listing primer and sequence (5′ → 3′) of *A2*,* IL-2R*,* IL-6F*,* IL-6R* and *IL-12R* which used in differential display PCR reaction.

Primers	Sequence 5′ → 3′	References
*A2*	5′-GAAACGGGTGGTGATCGC-3′	^97^
*IL-2R*	5′-AACCTTGGGCATGTAGAAGT-3′	^98^
*IL-6F*	5′-CCAGGATCCCAGCTATGAACTCCCTCT TC-3′	^99^
*IL-6R*	5′-GGAGAATTCGCTACTTCATCCGAATGACTC-3′	
*IL-12R*	5′-CTACGAAGAACTCAGATAG-3′	^100^

*A2* RAPD arbitrary primer, *IL-2* Interleukin-2, *IL-6* Interleukin-6, *IL-12* Interleukin-12, *Ref* References.

#### Real-time quantitative PCR assay

In this study, five primers for *TNF-α*^13^, *TNF-β*^101^, *IL-2*^98^, *IL-6*^99^ and *IL-12* genes^100^ were used with *β-actin*^102^ as housekeeping (standard) Table 6. The Real-Time PCR reaction mixture contains: 10 µl SYBR Green, 1 µl of 10 pm/µl forward primer,1 µl of 10 pm/µl reverse primer, 1 µl of cDNA (50 ng), and the volume completed up to 20 μL with sterile dH_2_O. The reaction conditions of the Real-Time PCR were as follows: initial denaturation at 95 °C for 10 min, 45 cycles of 95 °C for 10 s, annealing at 60 °C for 20 s, and elongation at 72 °C for 20 s. Data acquisition was performed during the extension step. This reaction analysis was performed by Rotor-Gene-6000-system analysis (Qiagen, USA). The difference in quantification ΔΔC_T_ cycle value (C_T_) of the average three replicates between the reference (C_T_ reference was used for all samples and the target (treated). The threshold of cycle equal of each identified gene was determined by automated threshold analysis on ABI System. The equations were calculated of the quantity gene expression according to Livak et al.^103^. The C_T_ value of each target gene was normalized to C_T (reference)_ to obtain ΔC_T (target)_ where:

$$\Delta {\text{C}}_{{{\text{T}}\,({\text{target)}}}} = ({\text{C}}_{{{\text{T}}\,({\text{target)}}}} - {\text{C}}_{{{\text{T}}\,({\text{reference}})}} )$$

$$\Delta {\text{C}}_{{{\text{T}}\,({\text{control}})}} = \, \left( {{\text{C}}_{{{\text{T}}\,({\text{control}})}} - {\text{C}}_{{{\text{T}}\,({\text{reference}})}} } \right)$$

**Table 6:** The specific primers of defence genes used in Real-Time PCR.

Target gene	Primers 5′ → 3′	Refernces
*β-actin* (standard)	F: 5′-ATGCCATTCTCCGTCTTGACTTG-3′	^102^
	R: 5′-GAACCTAAGCCACGATACCA-3′	
*TNF-α*	F: 5′-CCACACCACGTTGAGGCAGATCA-3′	^13^
	R: 5′-CCTTGACCGCTTCTCCACTCCA-3′	
*TNF-β*	F: 5′- GGTGCCCAGAGATGGCTTGTA-3′	^101^
	R: 5′-TTGTGTGGATTGATGAGAGGAGAGT-3′	
*IL-2*	F: 5′-AAGAGTCATCAGAAGAGGAAA-3′	^98^
	R: 5′-AACCTT GGGCATGTAGAAGT-3′	
*IL-6*	F: 5′-CCAGGATCCCAGCTATGAACTCCCTCTTC-3′	^99^
	R: 5′-GGAGAATTCGCTACTTCATCCGAATGACTC-3′	
*IL-12*	F: 5′-CACCACCTGCCCCACCTCAG-3′	^100^
	R: 5′-CTACGAAGAACTCAGATAG-3′	

*β-actin* Beta-actin, *TNF-α* Tumour Necrosis Factor-α, *TNF-β* Tumour Necrosis Factor-β, *IL-2* Interleukin-2, *IL-6* Interleukin-6, *IL-12* Interleukin-12 *Ref* References.

The quantity of ΔΔC_T_ to the target gene was calculated with

ΔΔC_T_ = (C_T (target) _− C_T (control)_) conferred to 2^-ΔΔCT^ algorithm.

### Data analysis

Data were analysed using the GLM procedure of SAS (2009)^104^ for testing the treatment effect. Besides, the significant differences between means were tested using Tukey's Studentized Range (HSD).

### Supplementary Information

Supplementary Figures.

## Data Availability

All data generated or analyzed during this study are included in this manuscript and its supplementary information files;
