# Supplementary material for: Exploring the impact of nano-Se and nano-clay feed supplements on interleukin genes, immunity and growth rate in European Sea Bass (Dicentrarchus labrax)
Source: Sci Rep. 2024 Feb 1;14:2631. doi: 10.1038/s41598-024-53274-y (PMC10834503; doi:10.1038/s41598-024-53274-y)
Supplement: Supplementary file 1 — Supplementary Figures. [file 41598_2024_53274_MOESM1_ESM.pdf]

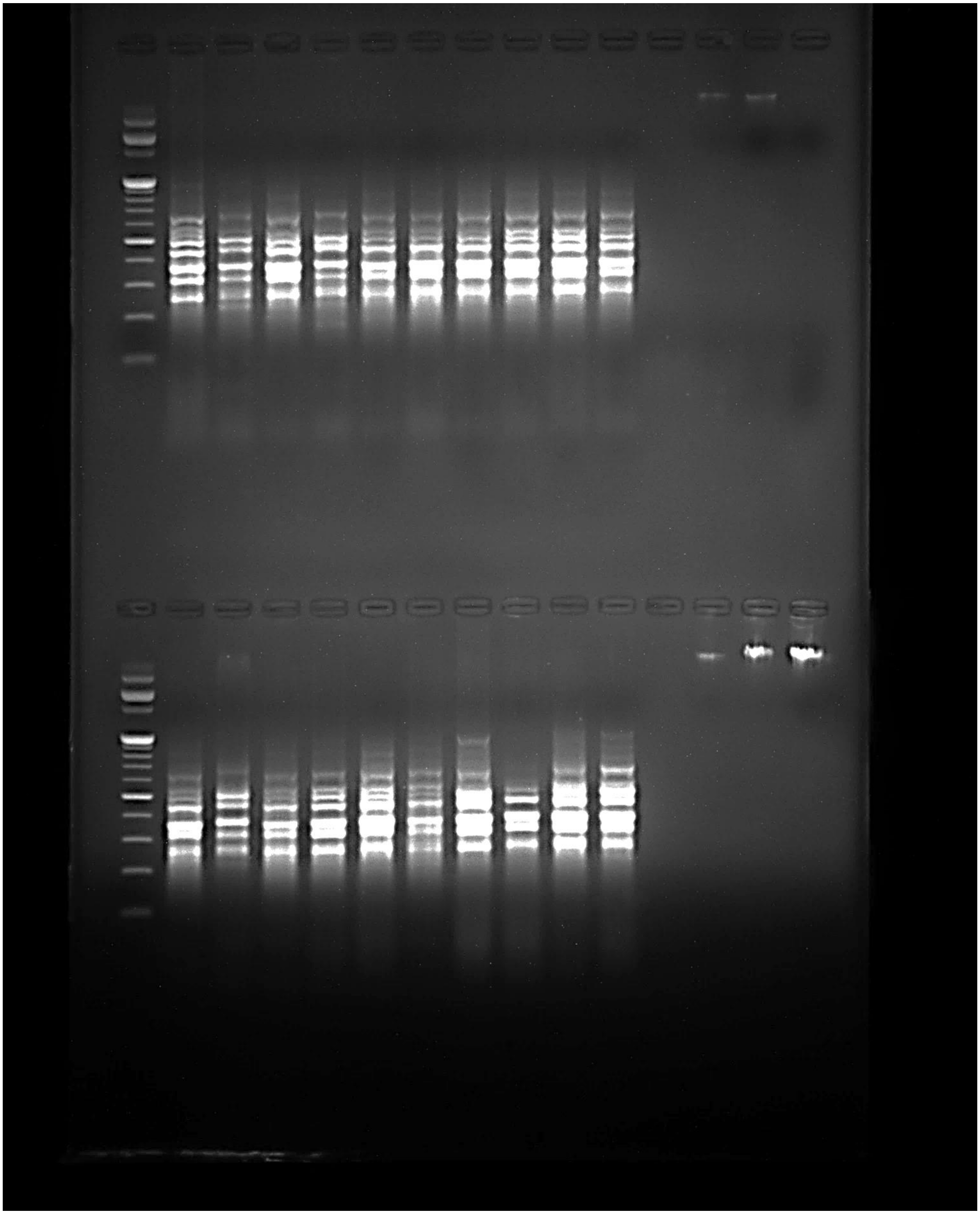

Fig. S1. DD- PCR for muscle tissue of fish fed on different concentrations of nano-clay (A) and nano-Se (B) using RAPD primer *A2*.

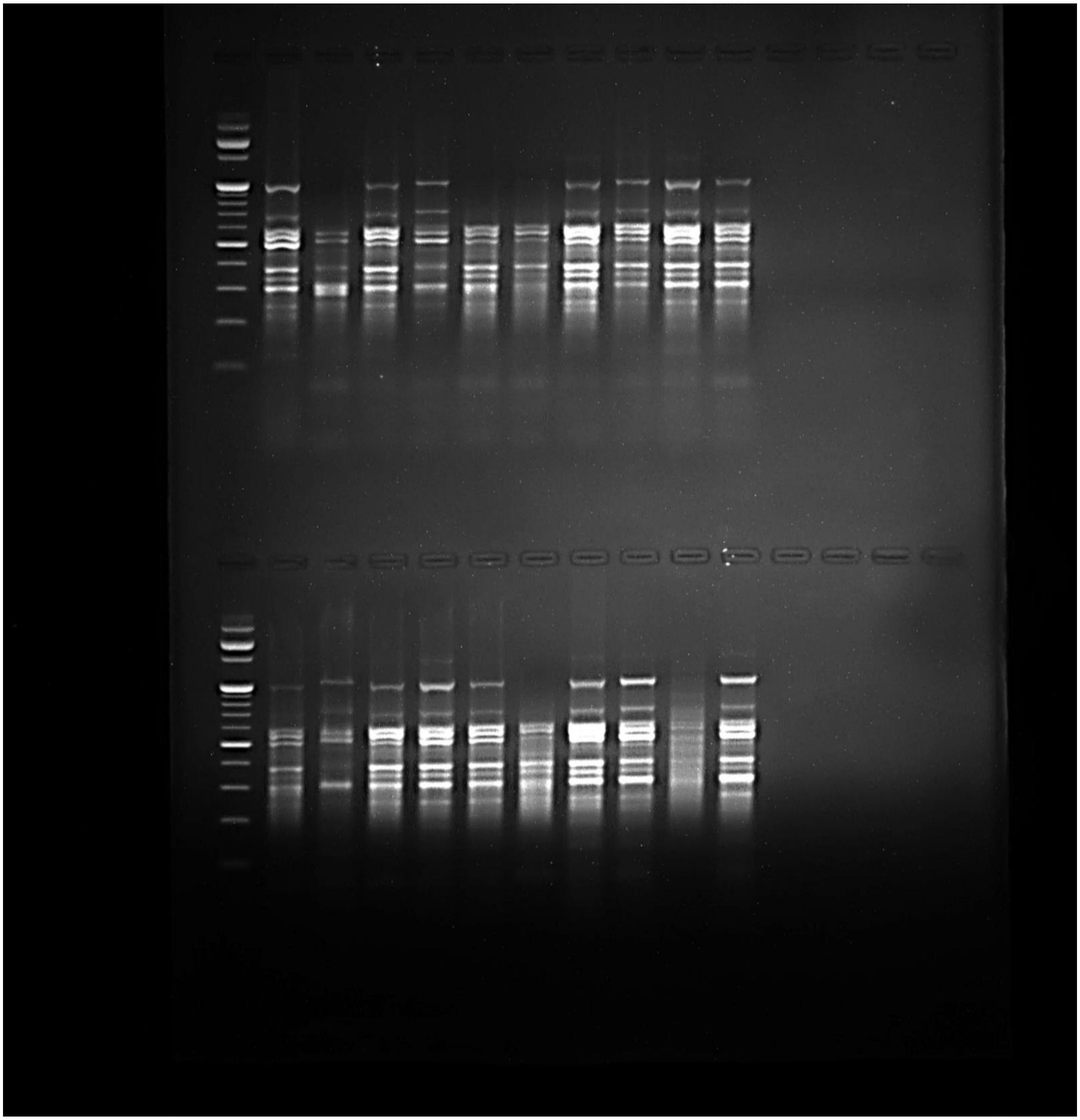

Fig. S2. DD-PCR for muscle tissue of fish fed on different concentrations of nano-clay (A) and nano-Se (B) using primer Interleukin-2 (*IL-2*).

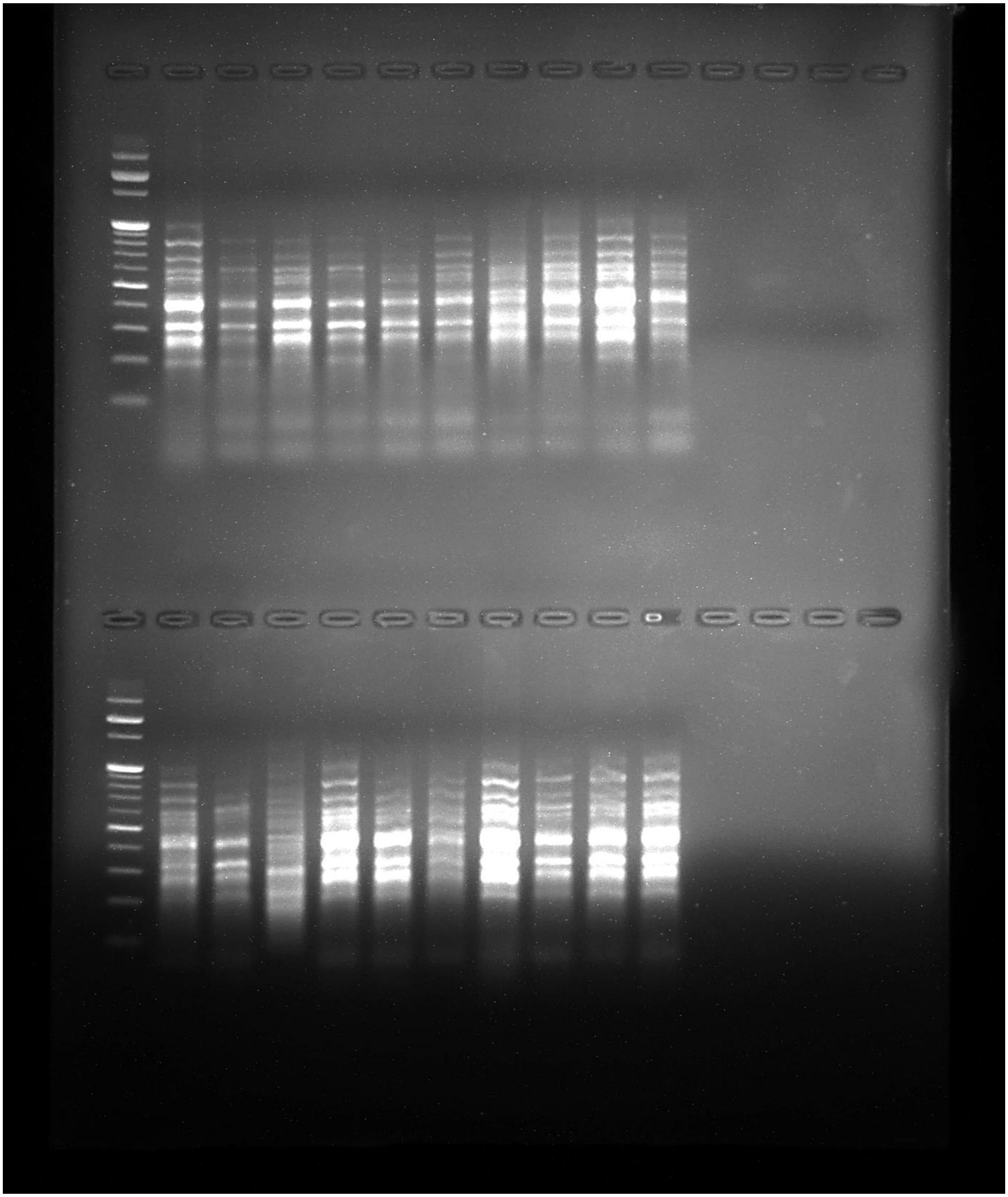

Fig. S3. DD- PCR for muscle tissue of fish fed on different concentrations of nano-clay (A) and nano-Se (B) using primer Interleukin-6F (*IL-6F*).

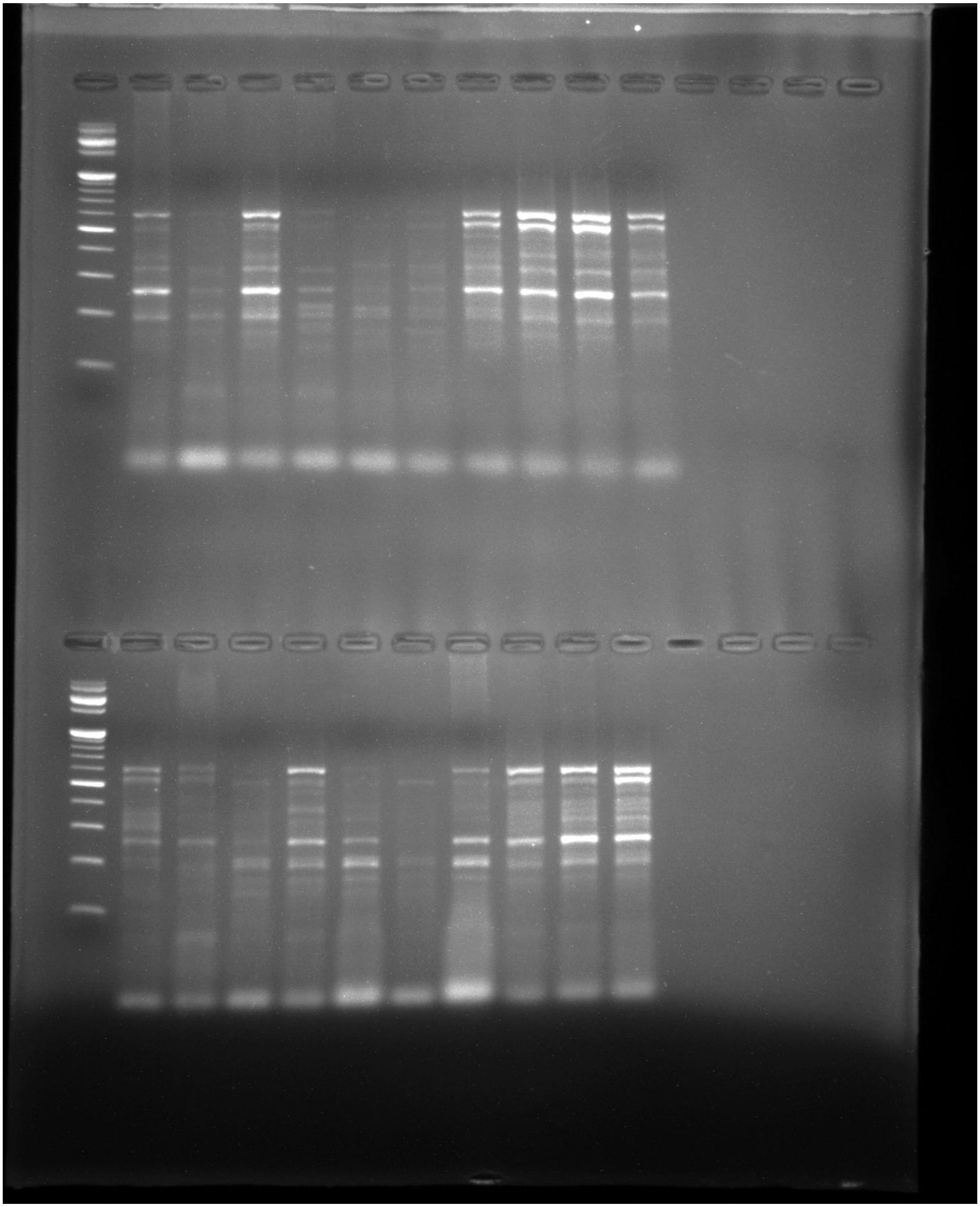

Fig. S4. DD-PCR for muscle tissue of fish fed on different concentrations of nano-clay (A) and nano-Se (B) using primer Interleukin-6R (*IL-6R*).

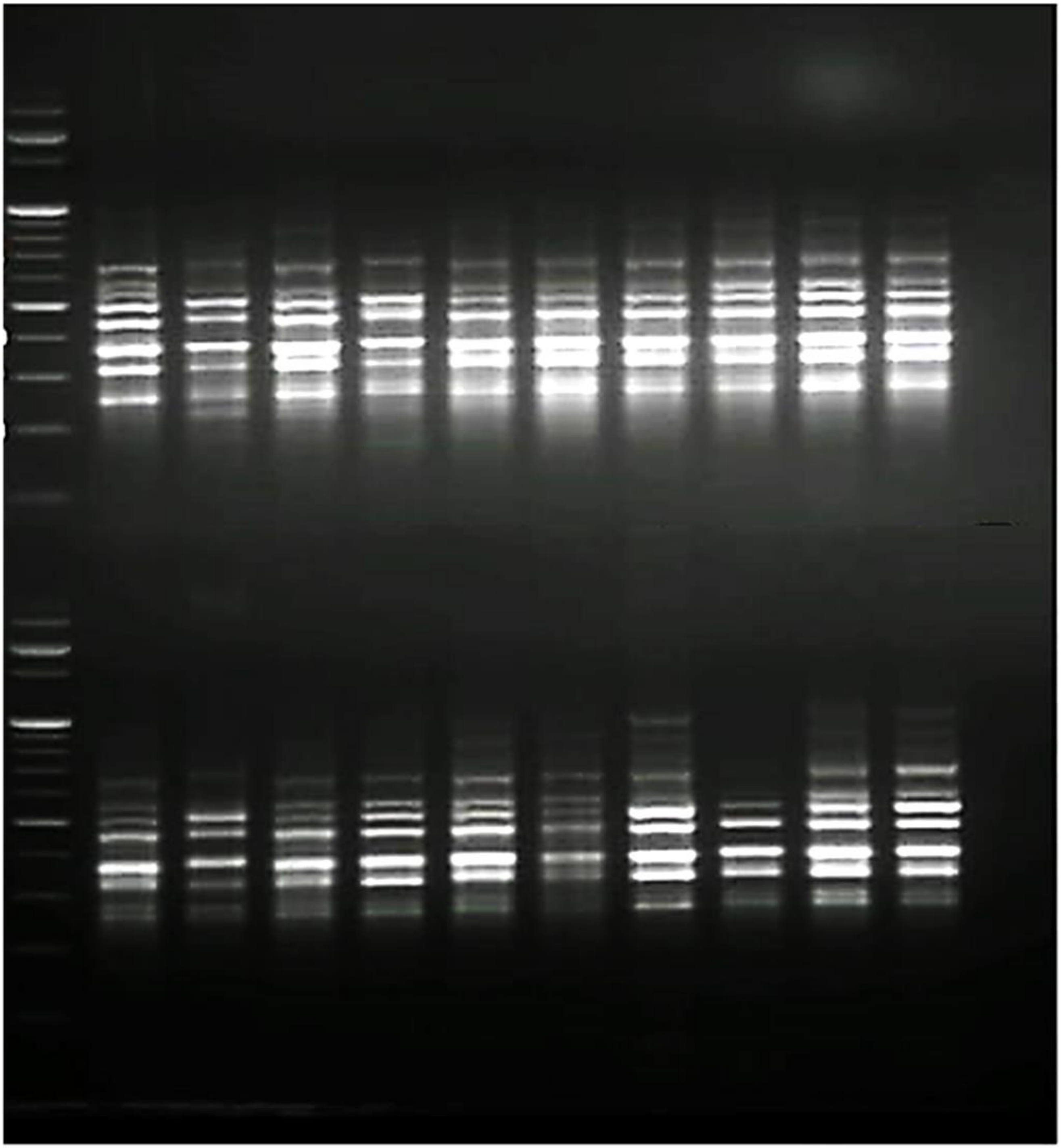

Fig. S5. DD- PCR for muscle tissue of fish fed on different concentrations of nano-clay (A) and nano-Se (B) using primer Interleukin-12R (*IL-12R*).
